# Genomic prediction with whole-genome sequence data in intensely selected pig lines

**DOI:** 10.1186/s12711-022-00756-0

**Published:** 2022-09-24

**Authors:** Roger Ros-Freixedes, Martin Johnsson, Andrew Whalen, Ching-Yi Chen, Bruno D. Valente, William O. Herring, Gregor Gorjanc, John M. Hickey

**Affiliations:** 1 The Roslin Institute and Royal (Dick) School of Veterinary Studies, The University of Edinburgh, Easter Bush, Midlothian, Scotland, UK; 2 Departament de Ciència Animal, Universitat de Lleida-Agrotecnio-CERCA Center, Lleida, Spain; 3 Department of Animal Breeding and Genetics, Swedish University of Agricultural Sciences, Uppsala, Sweden; 4 The Pig Improvement Company, Genus Plc, Hendersonville, TN USA

## Abstract

**Background:**

Early simulations indicated that whole-genome sequence data (WGS) could improve the accuracy of genomic predictions within and across breeds. However, empirical results have been ambiguous so far. Large datasets that capture most of the genomic diversity in a population must be assembled so that allele substitution effects are estimated with high accuracy. The objectives of this study were to use a large pig dataset from seven intensely selected lines to assess the benefits of using WGS for genomic prediction compared to using commercial marker arrays and to identify scenarios in which WGS provides the largest advantage.

**Methods:**

We sequenced 6931 individuals from seven commercial pig lines with different numerical sizes. Genotypes of 32.8 million variants were imputed for 396,100 individuals (17,224 to 104,661 per line). We used BayesR to perform genomic prediction for eight complex traits. Genomic predictions were performed using either data from a standard marker array or variants preselected from WGS based on association tests.

**Results:**

The accuracies of genomic predictions based on preselected WGS variants were not robust across traits and lines and the improvements in prediction accuracy that we achieved so far with WGS compared to standard marker arrays were generally small. The most favourable results for WGS were obtained when the largest training sets were available and standard marker arrays were augmented with preselected variants with statistically significant associations to the trait. With this method and training sets of around 80k individuals, the accuracy of within-line genomic predictions was on average improved by 0.025. With multi-line training sets, improvements of 0.04 compared to marker arrays could be expected.

**Conclusions:**

Our results showed that WGS has limited potential to improve the accuracy of genomic predictions compared to marker arrays in intensely selected pig lines. Thus, although we expect that larger improvements in accuracy from the use of WGS are possible with a combination of larger training sets and optimised pipelines for generating and analysing such datasets, the use of WGS in the current implementations of genomic prediction should be carefully evaluated against the cost of large-scale WGS data on a case-by-case basis.

**Supplementary Information:**

The online version contains supplementary material available at 10.1186/s12711-022-00756-0.

## Background

Whole-genome sequence (WGS) data have the potential to empower the identification of causal variants that underlie quantitative traits and diseases [1–4], increase the precision and scope of population genetic studies [5, 6], and enhance livestock breeding. Genomic prediction has been successfully implemented in the main livestock species and it has increased the rate of genetic gain [7]. Genomic prediction has provided multiple benefits, including greater accuracies of genetic evaluations in livestock populations, such as cattle and pig, and reduced generational intervals, most notably in dairy cattle. Since its early implementations, genomic prediction is typically performed using marker arrays that capture the effects of the (usually unknown) causal variants via linkage and linkage disequilibrium [8, 9]. In contrast, WGS data are assumed to contain the causal variants. For this reason, it was hypothesized that WGS could improve the accuracy of genomic predictions and its persistence across generations and breeds. Indeed, early simulations indicated that the inclusion of causal mutations based on WGS data could increase prediction accuracy. One simulation study indicated that the improvement in prediction accuracy relative to dense marker arrays ranged from 2.5 to 3.7% and persisted even when the training and testing sets were 10 generations apart [10]. Another study reported improvements in prediction accuracy of up to 30% when causal variants with a low minor allele frequency were captured by the WGS data [11]. However, benefits could be low in typical livestock populations due to small effective population sizes and recent directional selection [12].

During the last few years, there have been several attempts at improving the accuracy of genomic prediction with WGS in the main livestock species. Empirical results have been ambiguous so far. When predicting genomic breeding values within a population, some studies found no relevant improvement in accuracy with WGS data compared to marker arrays [13–16]. Other studies have found small but often unstable improvements (e.g., from 1 to 5% or no improvement depending on the prediction method used [17–19], or trait-dependent results [19, 20]). For genomic prediction across populations, the identification and inclusion of causal variants from WGS have been shown to improve prediction accuracy [21–24], especially for numerically small populations and for populations that are not represented in the training set [21, 23–27].

One of the most successful strategies to exploit WGS data consists of augmenting available marker arrays with preselected variants from WGS data based on their association with the trait of interest [28–31]. In some cases, this strategy improved the accuracy of genomic prediction by up to 9% (using a multi-breed training set) [30], and 11% (within line) [31], but this strategy did not improve prediction accuracy in other cases [15]. Nevertheless, these examples indicate that identifying causal variants from WGS data could enhance genomic prediction. Whole-genome sequence data has already been applied in genome-wide association studies (GWAS) to identify variants associated with a variety of traits in livestock [2, 32–34], including pigs [35, 36]. However, the fine-mapping of causal variants remains challenging due to the pervasive long-range linkage disequilibrium across extremely dense variants [37].

Accurate estimation of allele substitution effects and, ideally, identification of causal variants among millions of other variants are important applications of WGS data in research and breeding. These require large datasets that can capture most of the genome diversity in a population. Low-cost sequencing strategies have been developed, which typically involve sequencing a subset of the individuals in a population at low coverage and then imputing WGS data for the remaining individuals. However, the cost of generating accurate WGS data at such a large scale and the large computational requirements for the analyses of the resulting datasets have limited the population sizes or number of populations that have been tested in previous studies. This hinders the comparison of prediction accuracies across studies, which differ widely in population structures, sequencing strategies, and prediction methodologies used. The largest studies in livestock on the use of WGS for genomic prediction to date have been performed in cattle, for which a large multi-breed reference panel is available from the 1000 Bull Genomes Project [2, 17, 32]. This reference panel has enabled the imputation of WGS data in many cattle populations. The lack of such reference panels hampers the potential of WGS data in other species, such as pigs [35].

We have previously described our approach to impute WGS data in large pedigreed populations without external reference panels [38]. Following that strategy, we generated WGS data for 396,100 pigs from seven intensely selected lines with diverse genetic backgrounds and numerical sizes. The objectives of this study were to use this large pig dataset to assess the benefits of using WGS data for genomic prediction compared to using commercial marker arrays, to identify scenarios in which WGS provides the largest advantage, and to identify potential pitfalls for its effective use.

## Methods

### Populations and sequencing strategy

We re-sequenced the whole genome of 6931 individuals from seven commercial pig lines (Genus PIC, Hendersonville, TN) with a total coverage of approximately 27,243×. Breeds of origin of the nine lines included Large White, Landrace, Pietrain, Hampshire, Duroc, and synthetic lines. The sequencing effort in each of the seven lines was proportional to population size. The number of pigs that were available in the pedigree of each line and the number of sequenced pigs, by coverage, are summarized in Table 1. Approximately 1.5% (0.9 to 2.1% in each line) of the pigs in each line were sequenced. Most pigs were sequenced at low coverage, with a target coverage of 1 or 2×, but a subset of the pigs was sequenced at a higher coverage of 5, 15, or 30×. Thus, the average individual coverage was 3.9×, but the median coverage was 1.5×. Most of the sequenced pigs were born during the 2008–2016 period. The population structure across the seven lines was assessed with a principal component analysis using the sequenced pigs and is shown in Additional file 1: Fig. S1.

**Table 1:** Numbers of pigs sequenced and imputed

Line	Individuals sequenced	Individuals sequenced by coverage				Individuals used in the analyses			
		1x	2x	5x	15–30x	Pedigree	LD	HD	Imputed
A	1856	1044	649	73	90	122,753	39,485	66,763	104,661
B	1366	685	545	44	92	88,964	39,110	38,763	76,230
C	1491	628	728	54	81	84,420	35,343	34,358	66,608
D	731	362	311	16	42	79,981	16,650	54,297	60,474
E	760	394	274	27	65	50,797	22,768	20,685	41,573
F	381	193	137	16	35	35,309	11,747	17,758	29,330
G	445	217	176	15	37	21,129	11,472	6661	17,224

*Pedigree* number of individuals included in the pedigree used for imputation, *LD* number of individuals genotyped with the low-density marker array, *HD* number of individuals genotyped with high-density marker arrays, *Imputed* number of individuals with imputed genotypes that remain after filtering out individuals with low predicted imputation accuracy

The sequenced pigs and their coverage were selected following a three-part sequencing strategy that was developed to represent the haplotype diversity in each line. First (1), sires and dams with the largest number of genotyped progeny were sequenced at 2× and 1×, respectively. Sires were sequenced at a greater coverage because they contributed with more progeny than dams. Then (2), the individuals with the greatest genetic footprint on the population (i.e., those that carry more of the most common haplotypes) and their immediate ancestors were sequenced at a coverage between 1× and 30× (AlphaSeqOpt part 1; [39]). The sequencing coverage was allocated with an algorithm that maximises the expected phasing accuracy of the common haplotypes based on the accumulated family information. Finally (3), pigs that carried haplotypes with a low accumulated coverage (below 10× across all sequenced individuals) were sequenced at 1× (AlphaSeqOpt part 2; [40]). Sets (2) and (3) were based on haplotypes inferred from marker array genotypes (GGP-Porcine HD BeadChip; GeneSeek, Lincoln, NE), which were phased using AlphaPhase [41] and imputed using AlphaImpute [42].

Most sequenced pigs, as well as pedigree relatives, were also genotyped with marker arrays, either at low density (15k markers) using the GGP-Porcine LD BeadChip (GeneSeek) or at high density (50k or 80k markers), using different versions of the GGP-Porcine HD BeadChip (GeneSeek). In our study we only used markers included in the 50k array, which is the latest version of the high-density array. Markers in the 15k array were nested within the 50k array and markers from the 80k array that were not included in the 50k array were discarded. The number of pigs that were genotyped at each density is summarized in Table 1. Quality control of the marker array data was based on the individuals genotyped at high density. Markers with a minor allele frequency lower than 0.01, a call rate lower than 0.80, or that showed a significant deviation from the Hardy–Weinberg equilibrium were removed, separately for each line. After quality control, 38,634 to 43,966 markers remained for each line.

### Sequencing and data processing

Tissue samples were collected from ear punches or tail clippings. Genomic DNA was extracted using Qiagen DNeasy 96 Blood & Tissue kits (Qiagen Ltd., Mississauga, ON, Canada). Paired-end library preparation was conducted using the TruSeq DNA PCR-free protocol (Illumina, San Diego, CA). Libraries for resequencing at low coverage (1 to 5×) were produced with an average insert size of 350 bp and sequenced on a HiSeq 4000 instrument (Illumina). Libraries for resequencing at high coverage (15 or 30×) were produced with an average insert size of 550 bp and sequenced on a HiSeq X instrument (Illumina). All libraries were sequenced at Edinburgh Genomics (Edinburgh Genomics, University of Edinburgh, Edinburgh, UK).

DNA sequence reads were pre-processed using the Trimmomatic software [43] to remove adapter sequences from the reads. The reads were then aligned to the reference genome *Sscrofa11.1* (GenBank accession: GCA_000003025.6) using the BWA-MEM algorithm [44]. Duplicates were marked with Picard (http://broadinstitute.github.io/picard). Single nucleotide polymorphisms (SNPs) and short insertions and deletions (indels) were identified with the variant caller GATK HaplotypeCaller (GATK 3.8.0) [45, 46], using default settings. Variant discovery was performed separately for each individual and then a joint variant set for all the individuals in each population was obtained by extracting the variant positions from all individuals.

Read counts supporting each allele were extracted directly from the aligned reads stored in the BAM files, using a pile-up function, to avoid biases towards the reference allele introduced by GATK when applied on low-coverage WGS data [47]. This pipeline uses the pysam software (version 0.13.0; https://github.com/pysam-developers/pysam), which is a wrapper around the htslib program and the samtools package [48]. Read counts were extracted for all biallelic variants, after filtering out variants found in less than three sequenced individuals and variants in potential repetitive regions (defined as variants that had mean depth values 3 times greater than the average realized coverage) with VCFtools [49]. This pipeline delivered a total of 55.6 million SNPs (19.6 to 31.1 million within each line) and 10.2 million indels (4.1 to 5.6 million within each line). A more complete description of the variation across the lines is provided in [50].

### Genotype imputation

Genotypes were jointly called, phased, and imputed for a total of 483,353 pedigree-related individuals using the ‘hybrid peeling’ method implemented in AlphaPeel [51, 52]. This method uses all available marker array and WGS data. Imputation was performed separately for each line using complete multi-generational pedigrees, with 21,129 to 122,753 individuals per line (Table 1). We have previously published reports on the accuracy of imputation in the same populations using this method [38]. The estimated average allele dosage correlation (correlation between the real genotype and the imputed allele dosage) by individual was 0.94 (median: 0.97) [38]. Individuals with a low predicted imputation accuracy were removed before further analyses. An individual was predicted to have a low imputation accuracy when it or all of its grandparents were not genotyped with a marker array or when it had a low degree of connectedness to the rest of the population (defined as the sum of coefficients of pedigree-based relationships between the individual and the rest of individuals). These criteria were based on the analysis of imputation accuracy in simulated and empirical data [38]. In total, genotype data on 396,100 individuals remained, with 17,224 and 104,661 individuals per line (Table 1). The expected average individual-wise dosage correlation of the remaining individuals was 0.97 (median: 0.98) [38]. We also excluded variants with a minor allele frequency lower than 0.023 from further analyses, because their estimated variant-wise dosage correlations were lower than 0.90 [38]. After imputation, 32.8 million variants (14.5 to 19.9 million within each line) remained for downstream analyses, of which 9.9 million segregated across all seven lines.

### Traits

We analysed phenotypic data on eight complex traits that are commonly included in selection objectives of pig breeding programmes: average daily gain (ADG), backfat thickness (BFT), loin depth (LD), average daily feed intake (ADFI), feed conversion ratio (FCR), total number of piglets born (TNB), litter weight at weaning (LWW), and return to oestrus within 7 days after weaning (RET, binary trait). Most pigs with records were born during the 2008–2020 period. Breeding values were estimated by line with a linear mixed model that included polygenic and non-genetic (as relevant for each trait) effects. Deregressed breeding values (dEBV) were derived from the estimated breeding values for each trait following the method of VanRaden et al. [53]. Only individuals that had an own phenotype for the trait were retained for further analyses. The numbers of records for each trait used in the analyses of each line are provided in Table 2.

**Table 2:** Number of phenotype records by trait and line for the training and testing datasets

Trait	Set	A	B	C	D	E	F	G
ADG	Training	77,811	54,709	48,219	45,693	31,918	24,046	13,479
	Testing	9435	8387	6977	4789	3019	1808	1572
BFT	Training	70,529	52,910	47,512	42,636	31,127	21,892	13,300
	Testing	8560	7957	6747	4301	2936	1602	1568
LD	Training	75,117	54,537	48,054	43,517	31,987	24,154	13,303
	Testing	9021	8415	6995	4411	3024	1807	1570
ADFI	Training	20,535	8866	8235	11,573	11,930	4000	4457
	Testing	1358	638	802	641	478	97*	364
FCR	Training	19,805	8572	7857	11,378	11,804	3900	4364
	Testing	1328	624	775	624	477	97*	360
TNB	Training	12,250	9315	8438	7700	5834	–	2865
	Testing	254	428	400	23*	125*	–	98*
LWW	Training	–	7884	6251	–	–	–	2505
	Testing	–	246	220	–	–	–	47*
RET	Training	–	5928	5496	–	–	–	1481
	Testing	–	332	282	–	–	–	70*

*ADG* average daily gain, *BFT* backfat thickness, *LD* loin depth, *ADFI* average daily feed intake, *FCR* feed conversion ratio, *TNB* total number of piglets born, *LWW* litter weight at weaning, *RET* return to oestrus 7 days after weaning

^*^Included in multi-line scenarios, but excluded in within-line scenarios because of the limited size of the testing set

### Training and testing sets

We split the individuals in each line into training and testing sets. The testing sets were defined as individuals from full-sib families from the last generation of the pedigree (i.e., individuals that did not have any progeny of their own). Only families with a minimum of five full-sibs were considered. The training set was defined as all individuals that had a pedigree coefficient of relationship lower than 0.5 with any individual in the testing set. This design was chosen to mimic a realistic situation in which breeding programmes evaluate selection candidates that are available in a selection nucleus at any given time.

To assess the effect of the size of the training set on prediction accuracy, we created training sets with a reduced number of phenotype records for the three largest lines for the three traits with the largest number of records. We did this by removing the oldest animals, such that approximately the most recent 10, 20, or 35 to 45 thousand phenotype records remained in each of the reduced training sets.

Due to the computational requirements of the analyses (mainly for the preselection of the WGS variants to be included in the prediction equations), we could not replicate every analysis to assess the variability of the results. However, we did perform replicated validation analyses in the largest, one intermediate, and the smallest lines for two traits with a large and small number of phenotype records. To do this, we randomly split the testing sets into five subsets, with each full-sib family represented exclusively in one of the subsets. Training sets for each replicate were defined as for the general case.

### Genome-wide association study

To provide an association-based criterion to preselect variants for genomic prediction, we performed a single-SNP GWAS for each trait and line. This step included only the individuals in the training set. We fitted a univariate linear mixed model that accounted for genomic relationships as:

$$\mathbf{y}={\mathbf{x}}_{i}{\upbeta }_{i}+\mathbf{u}+\mathbf{e},$$

where $\mathbf{y}$ is the vector of dEBV, ${\mathbf{x}}_{i}$ is the vector of genotypes for the $i$th variant coded as 0 and 2 if homozygous for either allele or 1 if heterozygous, ${\upbeta }_{i}$ is the allele substitution effect of the $i$th variant on the trait, $\mathbf{u}\sim N(0,\mathbf{K}{\upsigma }_{\mathrm{u}}^{2})$ is the vector of polygenic effects with covariance matrix equal to the product of the polygenic additive genetic variance ${\upsigma }_{\mathrm{u}}^{2}$ and a genomic relationship matrix $\mathbf{K}$, and $\mathbf{e}$ is a vector of uncorrelated residuals. Due to computational limitations, the genomic relationship matrix $\mathbf{K}$ was calculated using only imputed genotypes from the marker array. We used the FastLMM software [54, 55] to fit the model.

### Within-line genomic prediction

To test whether variants from the WGS data could provide greater genomic prediction accuracy than the marker array data, we tested genomic prediction using variants from the marker array, from the WGS, or from both. The marker array data (also referred to as ‘Chip’) was set as the benchmark for prediction accuracy. It contained all ~ 40k variants in the marker array. For WGS, we preselected sets of variants because currently available methods for genomic prediction are not yet capable of handling datasets as large as the complete WGS without exorbitant computational resources. We tested different alternative strategies for preselecting variants for the prediction model based on the GWAS results:

1. Top40k: To mimic the number of variants in ‘Chip’, we preselected the variants with the lowest p-value (not necessarily below the significance threshold) in each of consecutive non-overlapping 55-kb windows along the genome. In addition, to test the impact of variant density on prediction accuracy, we preselected the top 10k, 25k, 75k, or 100k variants based on the same criterion.
2. ChipPlusSign: Only significant variants (p ≤ 10^–6^) were preselected from the WGS data and merged with those in ‘Chip’. When a 55-kb window contained more than one significant variant, only the variant with the lowest p-value was selected as a proxy to reduce the preselection of multiple significant variants that tag the same causal variant. When a significant variant from WGS data was already included in the marker array, it was considered only once and in the rare cases of genotype discordance between the WGS and marker array data, the genotype was replaced with the mean genotype value in that line. On average, 309 significant variants were identified per trait and line (range: 23 to 1083; Table 3) and merged with those in ‘Chip’.

**Table 3:** Number of significant variants from the whole-genome sequence data that were added to the marker array to create the ChipPlusSign set of variants

Trait	A	B	C	D	E	F	G	Multi-line
ADG	646	581	424	498	279	219	143	4731
BFT	1083	758	664	518	1030	218	237	6149
LD	633	579	458	518	222	215	43	7247
ADFI	145	224	169	23	183	–	119	767
FCR	198	224	162	95	56	–	134	1369
TNB	71	117	161	–	–	–	–	248
LWW	–	32	73	–	–	–	–	480
RET	–	184	31	–	–	–	–	60

*ADG* average daily gain, *BFT* backfat thickness, *LD* loin depth, *ADFI* average daily feed intake, *FCR* feed conversion ratio, *TNB* total number of piglets born, *LWW* litter weight at weaning, *RET* return to oestrus 7 days after weaning

Genomic prediction was performed by fitting a univariate model with BayesR [56, 57], which uses a mixture of normal distributions as the prior for variant effects, including one distribution that sets the variant effects to zero. The model was:

$$\mathbf{y}=\mathbf{1}\upmu +\mathbf{X}{\varvec{\upbeta}}+\mathbf{e},$$

where $\mathbf{y}$ is the vector of dEBV, $\bf 1$ is a vector of ones, $\upmu$ is the general mean, $\mathbf{X}$ is a matrix of variant genotypes, ${\varvec{\upbeta}}$ is a vector of variant effects, and $\mathbf{e}$ is a vector of uncorrelated residuals. The prior variance of the variant effects in ${\varvec{\upbeta}}$ had four components with means equal to zero and variances ${\upsigma }_{1}^{2}=0$, ${\upsigma }_{2}^{2}=0.0001{\upsigma }_{\mathrm{g}}^{2}$, ${\upsigma }_{3}^{2}=0.001{\upsigma }_{\mathrm{g}}^{2}$, and ${\upsigma }_{4}^{2}=0.01{\upsigma }_{\mathrm{g}}^{2}$, where ${\upsigma }_{\mathrm{g}}^{2}$ is the total genetic variance. We used a uniform and almost uninformative prior for the mixture distribution with the total genetic variance re-estimated in every iteration. We used a publicly available implementation of BayesR (https://github.com/syntheke/bayesR; accessed on 30 April 2021), with default settings. Prediction accuracy was calculated in the testing set as the correlation between the predicted genomic breeding values and the dEBV. Bias (inflation/deflation) of the prediction accuracy was calculated as the regression coefficient of the dEBV on the predicted genomic breeding values. For ease of comparison between traits and lines, the difference between prediction accuracy based on WGS and marker array data was calculated and analysed by fitting linear models with the size of the training set as a covariate and trait and line as fixed effects when appropriate.

### Multi-line genomic prediction

We considered multi-line scenarios in which the training set was formed by merging the training sets that had been defined for each line. All analyses were performed as for the within-line scenarios but with line as an additional effect in the prediction model. In the multi-line scenarios, the benchmark genomic prediction was obtained using all variants from the marker array that passed quality control and were imputed for at least one line (referred to as ‘ML-Chip’). For ease of computation, the strategies for preselection of variants from WGS were applied only to the subset of 9.9 million variants that had been called and imputed in all seven lines. Thus, we defined the variant sets ‘ML-Top40k’ and ‘ML-ChipPlusSign’ by preselecting variants following the same criteria as in the within-line scenarios, but using a multi-line GWAS with line as an additional effect. For ML-ChipPlusSign, 60 to 7247 significant variants were identified per trait (Table 3) and merged with those in ML-Chip. For comparison purposes, genomic prediction accuracy was calculated separately for the testing set of each line.

## Results

### Within-line genomic prediction accuracy

**Fig. 1:**
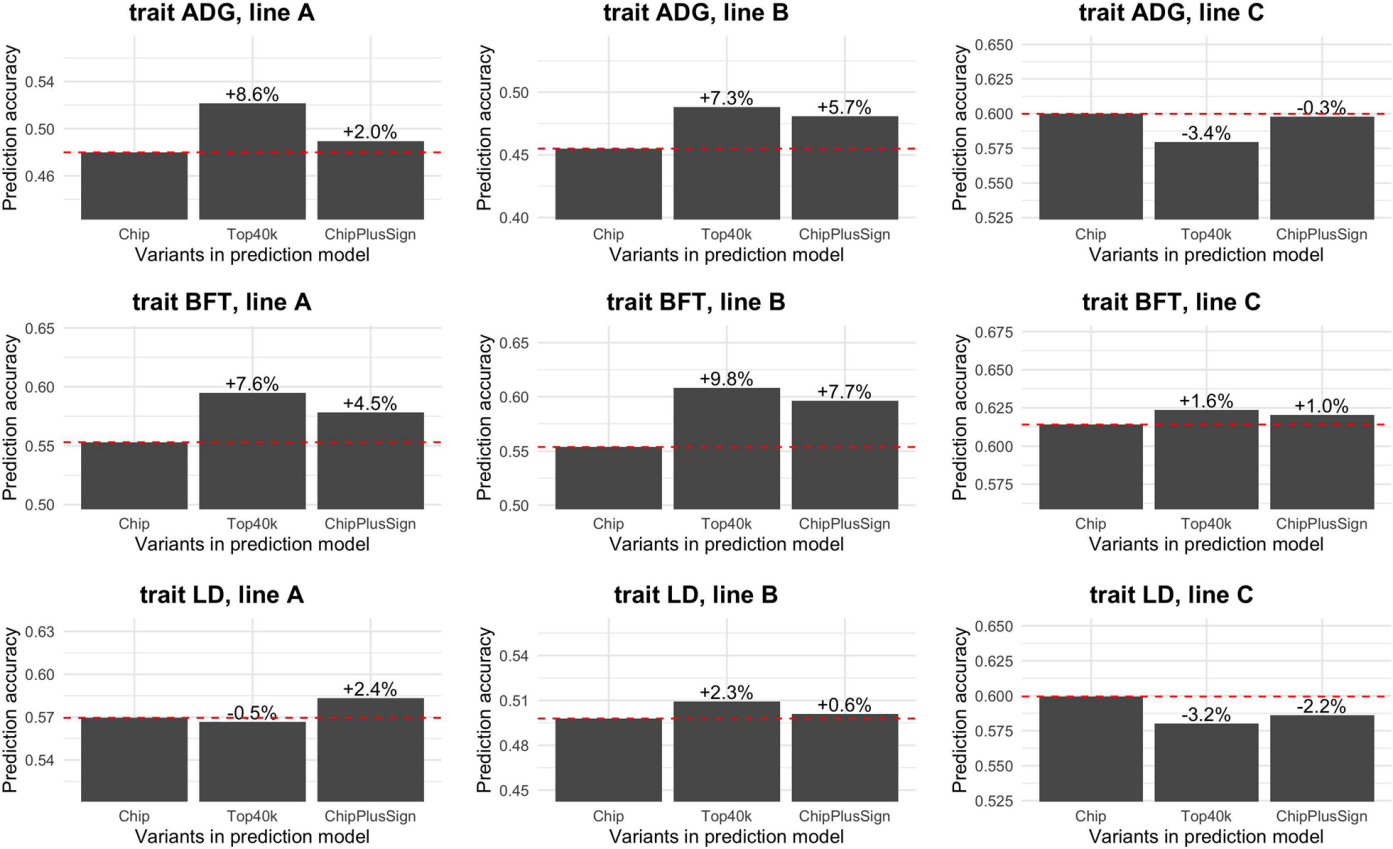
Genomic prediction accuracy for each set of variants for the ADG, BFT, and LD traits in the three largest lines. Dashed line at value of marker array (Chip) as a reference. Values indicate relative differences to marker array

Whole-genome sequence data improved genomic prediction accuracy compared to marker array data in some scenarios, especially when there was a sufficiently large training set and when an appropriate set of WGS variants was preselected. Figure 1 shows the prediction accuracy for the three traits and three lines with the largest training sets using the two different sets of WGS variants. Results for the rest of traits and lines, as well as results for the bias, are in Additional file 2: Fig. S2. For BFT in line B, compared to the marker array results, the two tested sets of WGS variants increased prediction accuracy by 0.054 (+9.8%) for Top40k and by 0.043 (+7.7%) for ChipPlusSign. However, the performance of WGS was not robust and differed by trait and line, and even between replicates for the same trait and line (see Additional file 3: Fig. S3 and Additional file 4: Table S1), often leading to no improvement in prediction accuracy or even reduced prediction accuracy relative to the marker array data. For instance, Top40k reduced prediction accuracy by 0.020 (–3.4%) for ADG in line C and ChipPlusSign reduced accuracy by 0.020 (–2.2%) for LD in line C. Using WGS data reduced bias compared to the marker array data in only some cases.

**Fig. 2:**
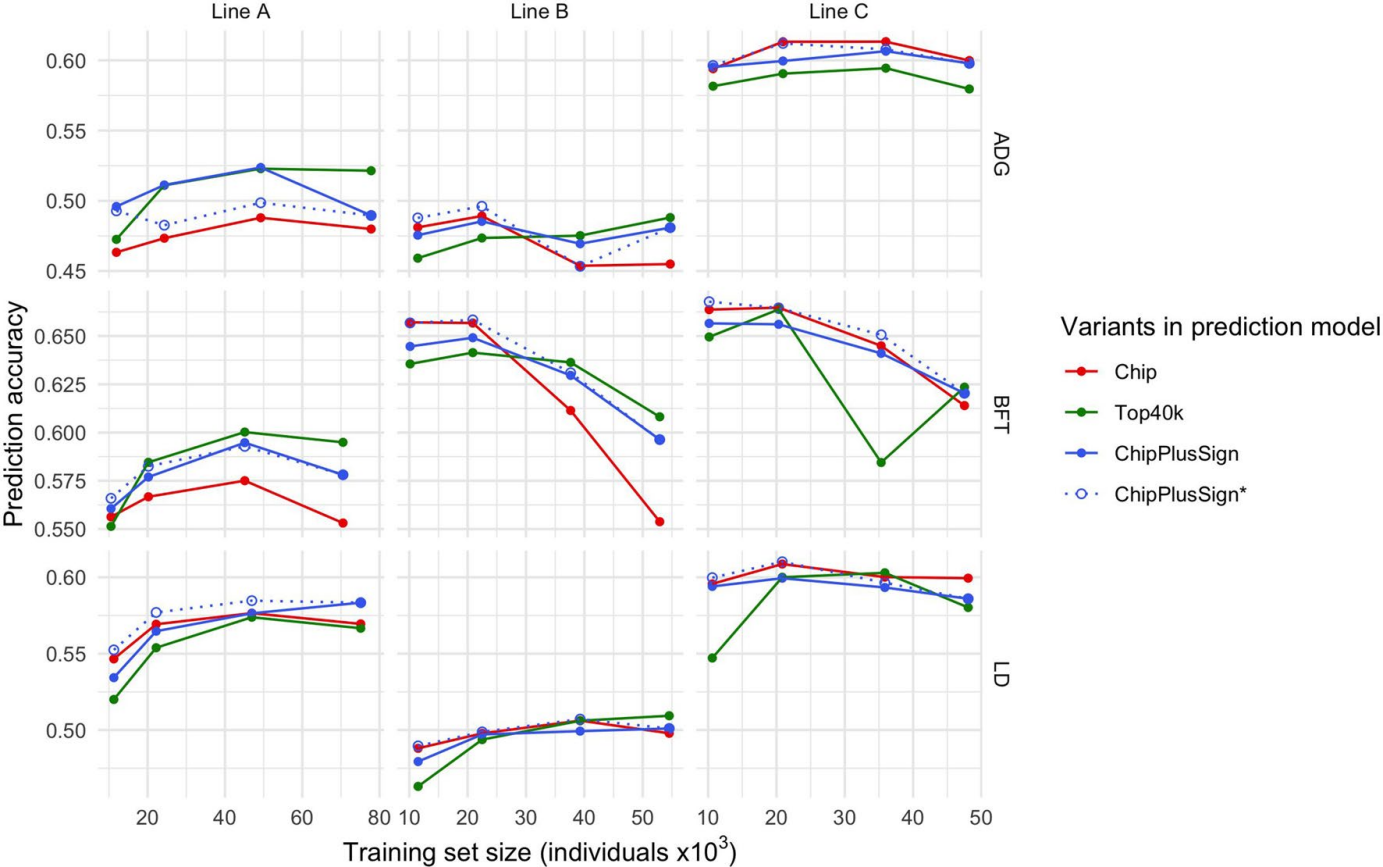
Genomic prediction accuracy with the marker array (Chip) or with preselected WGS data (Top40k, ChipPlusSign, and ChipPlusSign*) with varying training set sizes for the ADG, BFT, and LD traits in the three largest lines. In ChipPlusSign*, variants are preselected based on associations tested using the largest training set available for each trait and line

**Fig. 3:**
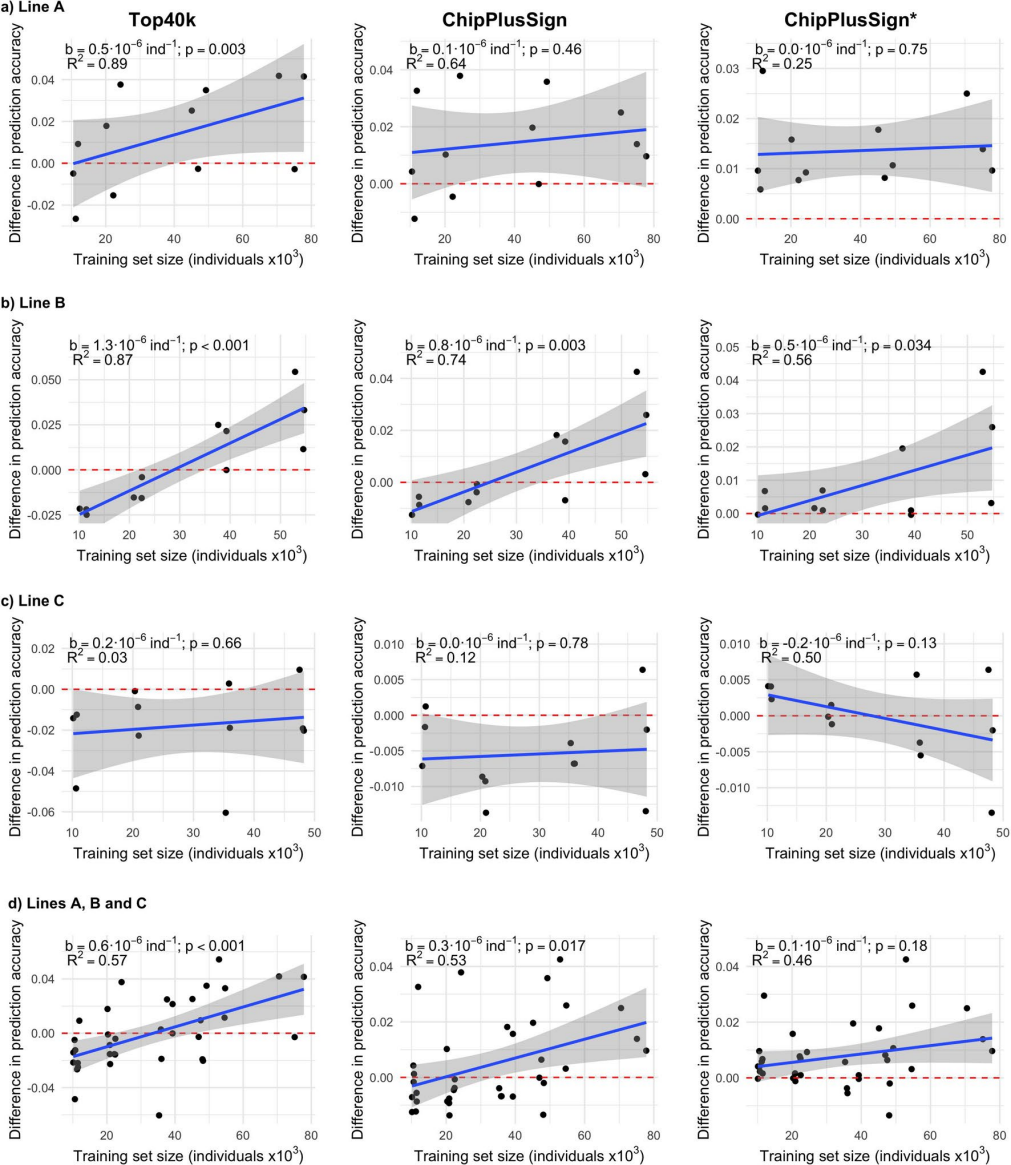
Effect of training set size on the genomic prediction accuracy for each set of preselected WGS variants in the three largest lines. The difference between each set of preselected WGS variants (Top40k, ChipPlusSign, and ChipPlusSign*) and the marker array (Chip) is shown. Red dashed line at ‘no difference’. Regression coefficient (b) and p-value of training set size is provided, as well as the coefficient of determination (R^2^) of the model. Results for traits ADG, BFT, and LD were jointly analysed, and the linear model included the trait effect (panels **a** to **c**) or the trait and line effects (panel **d**)

Results within trait and line (Fig. 2) confirmed that the impact of the use of WGS data on genomic prediction accuracy depends on the line, but also showed that the capacity of WGS variants to improve the genomic prediction accuracy compared to those of the marker arrays tended to increase with size of the training set. Jointly analysing the results for ADG, BFT, and LD (Fig. 3), we found significant positive regression coefficients of the difference in prediction accuracy based on WGS variants versus marker array variants on training set size in lines A (only Top40k) and B (all sets of WGS variants) but not in line C, for which the regression coefficient was not statistically significant for any set of WGS variants. When computed considering the three lines jointly (Fig. 3d), the regression coefficient of the difference in prediction accuracy on the training set size was 0.6 × 10^–6^ per individual (p < 0.001) for Top40k and 0.3·10^–6^ per individual (p = 0.017) for ChipPlusSign. This was, at least partly, driven by the smaller number of significant associations that were detected with smaller training sets. For ChipPlusSign, with a training set of 20k individuals or less, 118 to 287 significant variants were added to the marker array; with a training set of 35k to 45k individuals, 288 to 709 significant variants were added; and with all available individuals in the training set, 424 to 1083 significant variants were added. Thus, if the marker array was augmented with the significant variants detected with all available individuals (ChipPlusSign*; Figs. 2 and 3), the use of WGS data yielded the same prediction accuracy than the marker array data or higher in most scenarios even when the set for training the prediction equation was smaller.

**Fig. 4:**
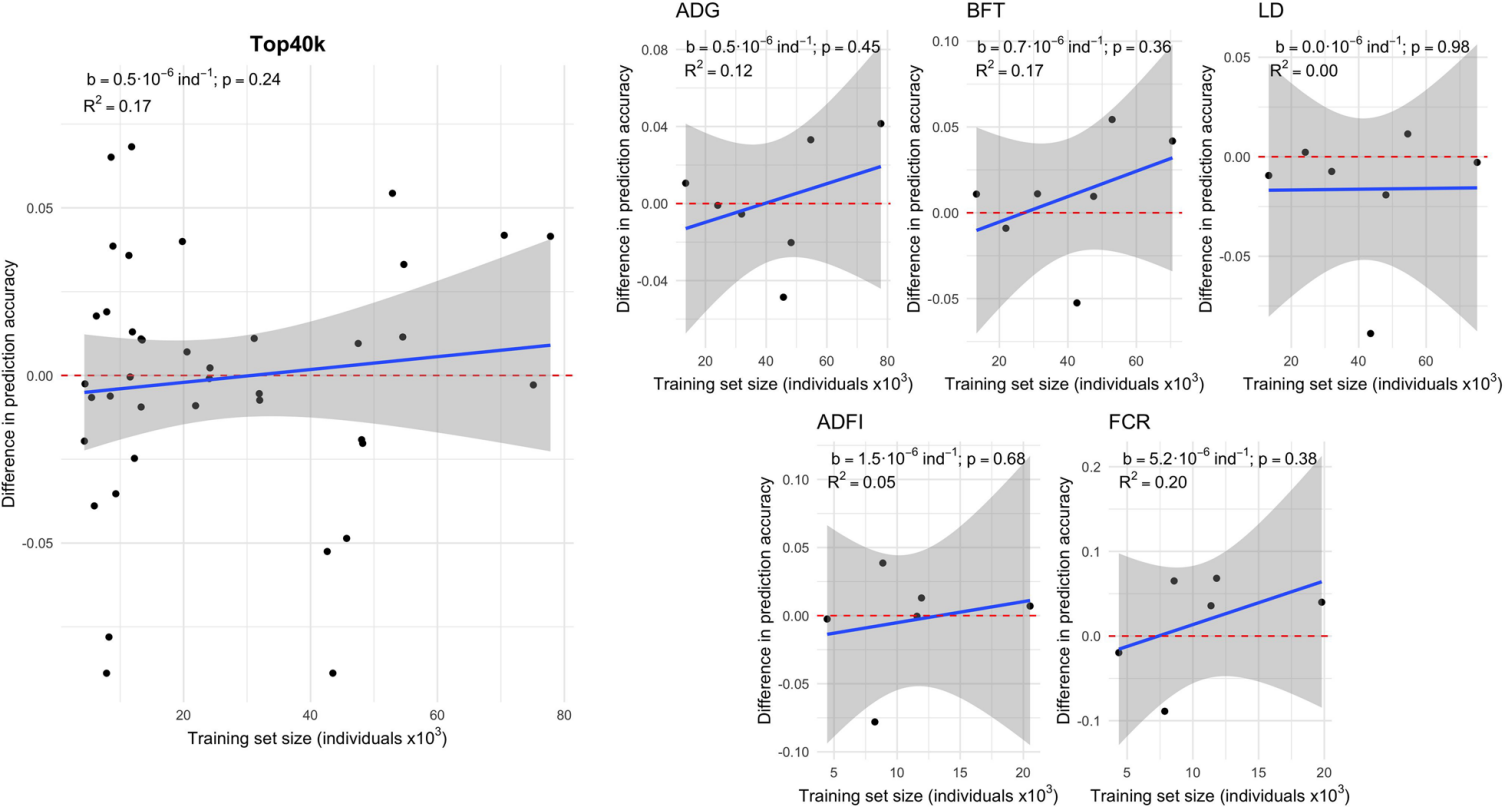
Genomic prediction accuracy based on the Top40k variants. The difference between the Top40k and marker array is shown for all traits and lines (left) or by trait (right). Red dashed line at ‘no difference’. Regression coefficient (b) and p-value of training set size is provided, as well as the coefficient of determination (R^2^) of the model. The linear model for the joint analyses included the trait effect

**Fig. 5:**
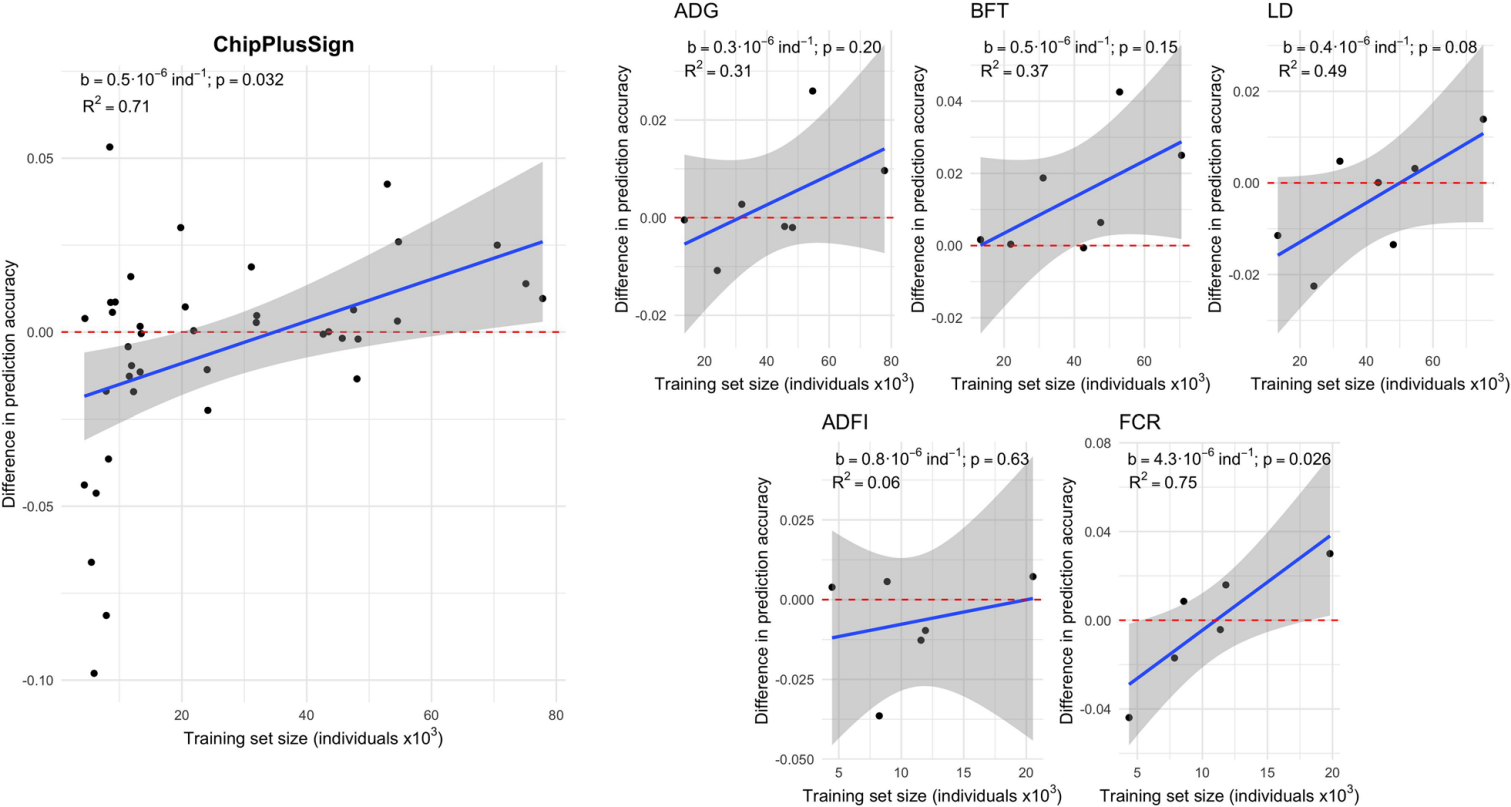
Genomic prediction accuracy based on the ChipPlusSign variants. The difference between the ChipPlusSign and marker array is shown for all traits and lines (left) or by trait (right). Red dashed line at ‘no difference’. Regression coefficient (b) and p-value of training set size is provided, as well as the coefficient of determination (R^2^) of the model. The linear model for the joint analyses included the trait effect

Figures 4 and 5 show the difference in prediction accuracy using Top40k and ChipPlusSign compared to using marker array variants against the size of the training set that was available for each trait in each of the lines. We observed large variability for the difference in prediction accuracy, especially when the training set was small. This variability was larger for Top40k than for ChipPlusSign, such that the shrinkage of the variation was more noticeable in ChipPlusSign as the training set size increased. Within trait and line, the variability across replicates was also larger for Top40k than for ChipPlusSign (see Additional file 4: Table S1). Gains in prediction accuracy were low-to-moderate in the most favourable cases. In the most unfavourable cases, we observed large losses in prediction accuracy for Top40k but more limited losses for ChipPlusSign with moderate training set sizes. When data from all traits and lines were jointly analysed, the regression coefficient of the difference in prediction accuracy on size of the training set was positive but had stronger statistical evidence for ChipPlusSign (b = 0.5 × 10^–6^ per individual; p = 0.032; R^2^ for each trait between 0.06 and 0.75) than for Top40k (b = 0.5 × 10^–6^ per individual; p = 0.24; R^2^ for each trait between 0.00 and 0.20).

We observed diminishing increases in accuracy when we increased the density of the variants used in prediction. Increasing the number of variants from 40k in Top40k to 75k yielded small improvements in genomic prediction accuracy compared to Top40k, but increases up to 100k variants provided smaller or null additional gains (see Additional file 5: Fig. S4).

### Multi-line genomic prediction accuracy

**Fig. 6:**
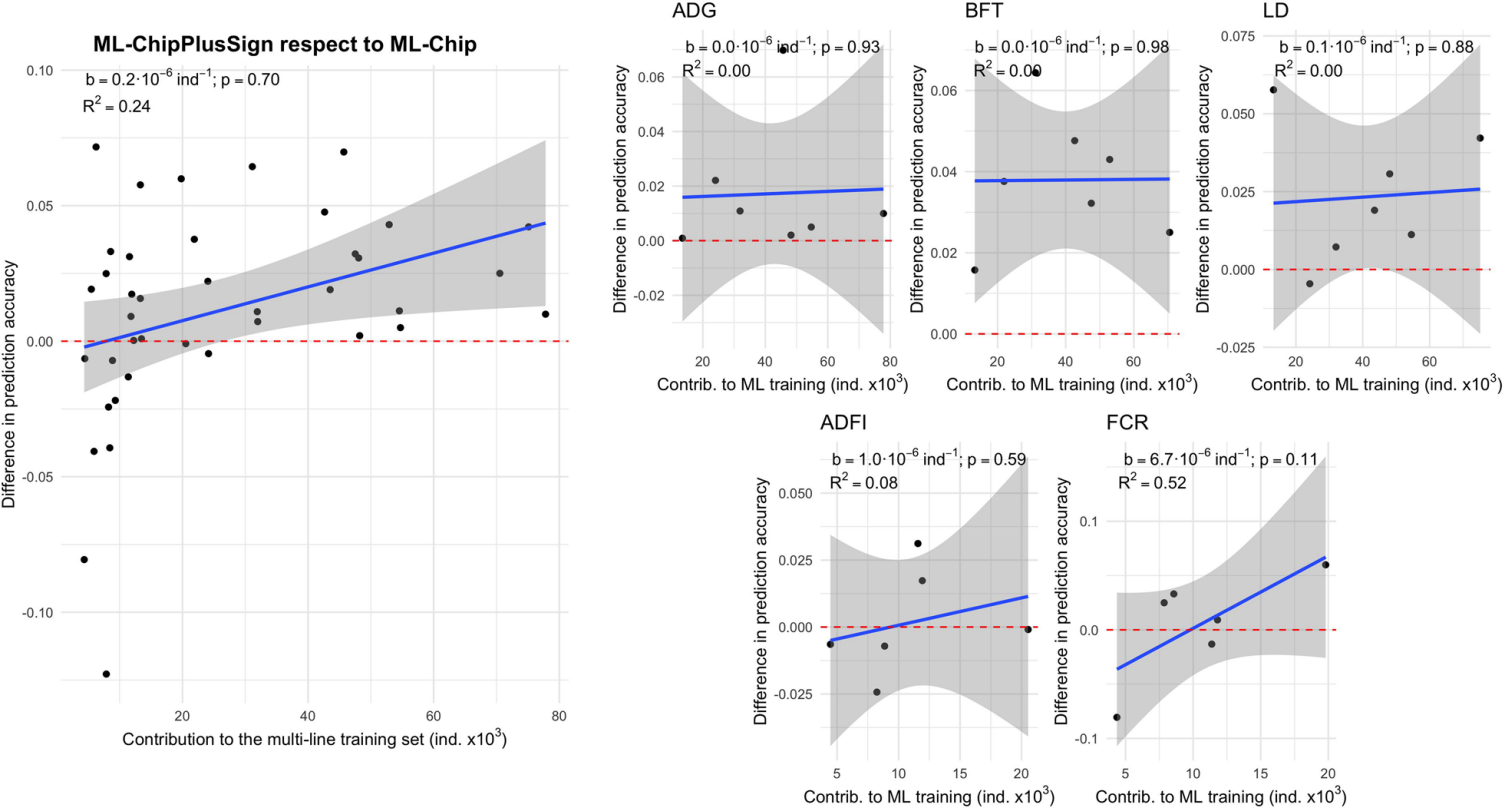
Genomic prediction accuracy based on the ML-ChipPlusSign variants. The difference between ML-ChipPlusSign and marker array (ML-Chip) is shown for all traits and lines (left) or by trait (right). Red dashed line at ‘no difference’. Regression coefficient (b) and p-value of training set size is provided, as well as the coefficient of determination (R^2^) of the model. The linear model for the joint analyses included the trait effect

**Fig. 7:**
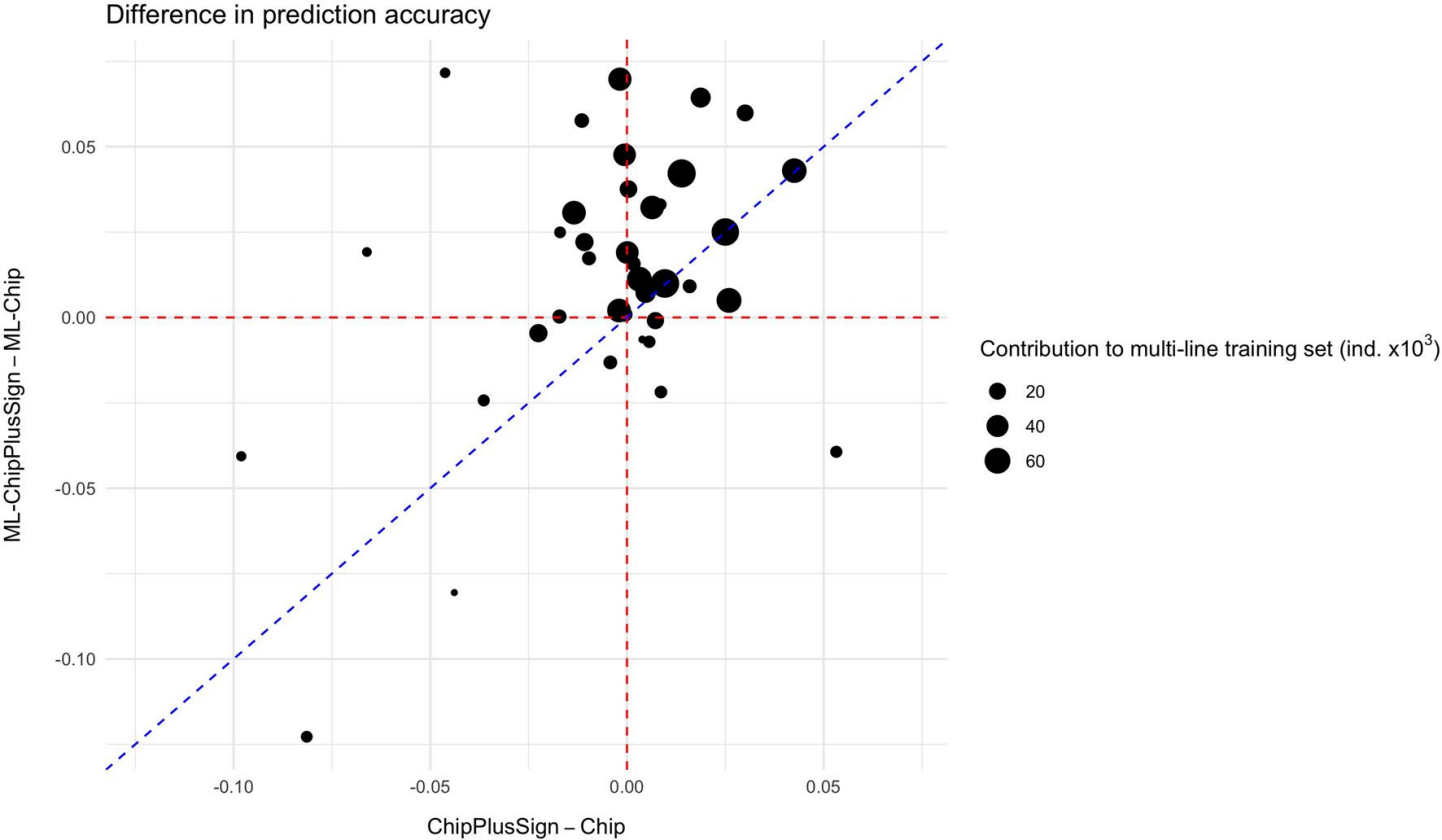
Comparison of the difference in genomic prediction accuracy in the multi-line scenarios (between ML-ChipPlusSign and ML-Chip) and in the within-line scenarios (between ChipPlusSign and Chip) for all traits and lines. Red dashed line at ‘no difference’. Blue dashed line is the bisector

The accuracy of genomic prediction trained across multi-line datasets was systematically lower than for the within-line genomic prediction scenarios (see Additional file 6: Fig. S5). Nonetheless, when using multi-line training sets, using the ML-ChipPlusSign variants in general increased genomic prediction accuracy relative to using the marker array variants (ML-Chip; Fig. 6). For the traits that accumulated the largest multi-line training sets (i.e., ADG, BFT, and LD), the improvements in prediction accuracy for each line seemed unrelated to the number of individuals that that line contributed to the multi-line training set. However, for traits that accumulated smaller multi-line training sets (i.e., ADFI and FCR), using ML-ChipPlusSign only improved prediction accuracy for the lines that contributed more individuals to the multi-line training set and reduced prediction accuracy for the lines that contributed less individuals to the multi-line training set. Therefore, as for the within-line scenarios, the greatest improvements in prediction accuracy from the use of WGS data were achieved for the largest lines, although using ML-ChipPlusSign in the multi-line scenarios also improved prediction accuracy compared to using ML-Chip for some traits and lines for which no improvements were observed in the within-line scenarios, including numerically small lines (Fig. 7). In contrast, results for ML-Top40k were not robust across traits (see Additional file 7: Fig. S6).

## Discussion

Our results showed that the use of WGS data has some potential to improve genomic prediction accuracy compared to marker arrays in intensely selected pig lines, but its use with current implementations of genomic prediction should be carefully evaluated. The small and non-robust improvements in accuracy that were observed indicate that the strategies for analysing the WGS data that we tested were likely suboptimal. The more favourable results for WGS variants in the largest training sets indicated that we might have not reached the critical mass of data that is needed to leverage the potential of WGS, especially in scenarios where genomic prediction with marker arrays is already yielding high accuracy. The results from several traits and lines with different training set sizes allowed us to identify the most favourable scenarios for genomic prediction with WGS. In the following, we will discuss (1) the prediction accuracy that we achieved with WGS data compared to the commercial marker array data and the scenarios in which WGS may become beneficial, (2) the potential pitfalls for the effective use of WGS for genomic prediction and the need for optimised pipelines for generating and analysing WGS datasets, and (3) the suitability of WGS data for genomic prediction with current implementations of genomic prediction.

### Prediction accuracy with whole-genome sequence data

We compared the genomic prediction accuracy based on the current marker array (Chip) with that based on sets of preselected WGS variants such that the number of variants remained similar across sets. Improvements in prediction accuracy are expected to be limited if current marker arrays are already sufficiently dense to capture a large proportion of the genetic diversity in intensely selected livestock populations because such populations typically have small effective population sizes [12, 17]. Nevertheless, modest improvements from the use of WGS data for genomic prediction within line have been achieved under certain scenarios across several studies [17–20, 29, 31]. In our study, the most robust results across traits and lines were obtained with the ChipPlusSign variant sets, where the marker array was augmented with WGS variants that had statistically significant associations to the trait. Results from simulated traits (see Additional file 8 and Additional file 9: Figs. S7 and S8) confirmed the greater robustness of genomic predictions based on ChipPlusSign compared to Top40k that was observed for the empirical traits. This is consistent with previous reports that showed an improvement in prediction accuracy under similar approaches for preselecting WGS variants [28–31].

Using ChipPlusSign, we augmented the marker array with 23 to 1083 significant variants preselected from WGS data across the different scenarios. In the most successful scenarios, a minimum of around 200 significant variants were added and prediction accuracy improved by 0.025 on average with training sets of around 80k individuals. Other studies suggested additions of a larger number of variants. In Hanwoo cattle, adding around 12k variants (3k with the lowest p-values for each of four traits) to a custom 50k marker array increased accuracy by up to ~ 0.06 [31]. In sheep, adding around 400 variants (preselected by GWAS as those that were significantly associated within windows identified by regional heritability mapping) to a 50k marker array increased accuracy by 0.09 [30]. Other studies expressed the results as reliabilities and also found improvements of reliability by adding larger numbers of variants to marker arrays. In Nordic cattle, adding 1623 variants (preselected by aggregating 3 to 5 tag variants for each of the top 5 to 15 quantitative trait loci (QTL) per trait and breed) to a 50k marker array increased reliability by up to 0.05 [28], but a similar approach produced negligible improvements for traits with low heritability [58]. In Holstein cattle, adding around 16k variants (preselected based on the size of allele substitution effect estimates) to a 60k marker array increased reliability on average by 0.027 (up to 0.048) [29].

The only modest increases in accuracy obtained with ChipPlusSign and Top40k compared to the marker array could also be a consequence of the difficulty for fine-mapping causal variants through GWAS on WGS data. Theoretically, inclusion of all causal variants associated with a trait in the marker array should improve genomic prediction accuracy [59]. Although WGS data allow the detection of a very large number of associations, problems such as false positives or p-value inflation also become more severe, so that the added noise might offset the detected signal. For instance, results in cattle showed that GWAS on WGS did not detect clearer associated regions than marker arrays and failed to capture QTL for genomic prediction [13], because the effects of potential QTL were spread across multiple variants. Genomic prediction using WGS variants that were preselected based on GWAS performed better for traits with simple genetic architectures based on results from simulated traits (see Additional file 9:Figs. S7 and S8), for which traits with a high heritability and small number of quantitative trait nucleotides (QTN) were more likely to show larger improvements in prediction accuracy when using WGS compared to marker arrays. This is consistent with expectations and previous simulation results [60] that indicated that the benefit of WGS for genomic prediction is limited by the number and size of QTN. Therefore, for largely polygenic traits (as most traits of interest in livestock production), training sets need to be very large before the use WGS data can increase genomic prediction accuracy [60].

The advantage of using WGS may also be limited by the small effective population size of livestock populations under selection [61] and by the current training set sizes, especially in scenarios where marker arrays are already yielding high genomic prediction accuracy [13, 18]. The use of WGS in multi-line training sets could be particularly beneficial because they allow a larger training set with a low level of pairwise relationships among individuals. Indeed, previous simulations suggested that WGS data might be the most beneficial with multi-breed reference panels [62], especially for numerically small populations. Our results with a multi-line training set indicated that WGS can improve prediction accuracy by up to 0.04. However, in general, the multi-line predictions were less accurate than those obtained for within-line scenarios. In our multi-line scenarios, we only used variants that segregated across all seven lines. We observed that population-specific variants accounted only for small fractions of the genetic variance [50] and it seems unlikely that they would contribute much to genomic prediction accuracy across breeds. Another possible reason for the lower accuracy of multi-line predictions is that allele substitution effects of the causal mutations may differ between lines. This can be caused by differences in allele frequencies, contributions of non-additive effects and different genetic backgrounds, or even gene-by-environment interactions, among others [22, 63].

We observed that the robustness of genomic prediction with WGS data across traits and lines was low, resulting in a drop in prediction accuracy in some scenarios. Previous studies have shown that using the same data for preselecting variants through GWAS and for training the prediction equation can reduce the accuracy of genomic predictions and bias the resulting predicted genomic breeding values [15, 64]. We observed no systematic increase in accuracy or bias when randomly splitting the training set into two distinct subsets, one for GWAS to preselect the predictor variants and the other for training the prediction equation (see Additional file 10: Fig. S9). One possible reason for this result is that the two subsets were not strictly independent because they belonged to the same population. The reduction in the number of individuals available for training may also have negatively affected genomic prediction accuracy.

We did not directly evaluate the persistence of the accuracy of genomic predictions across generations, but previous studies with empirical data found that the use of WGS data did not increase the persistence of accuracy [13]. We expect that persistence of accuracy will only improve when causal variants can be successfully identified and their non-additive effects are accounted for.

### Potential pitfalls for the effective use of WGS for genomic prediction

Effective use of WGS for genomic prediction can only be achieved when many other steps are completed to produce genotype data at the whole-genome level. Each of these steps has potential pitfalls that can limit the success of using WGS. This includes optimization of the choice of individuals to sequence, of the bioinformatic pipeline to call variants, of the imputation of the WGS, and of filtering of variants. When combined with the multiplicity of strategies to preselect variants for genomic prediction (which is unavoidable with current datasets, genomic prediction methods, and computational resources), the whole process includes many variables that can affect the final result and that are not yet well understood. Therefore, a much greater effort for optimising the whole process is required. Here, we tested relatively simple approaches to evaluate how they performed with large WGS datasets. We have previously discussed what, in our opinion, are the main pitfalls of our approach for selection of individuals to sequence [52] and the biases that may appear during processing of sequencing reads [47]. Here, we will focus our discussion on imputation of WGS data and its use for genomic prediction.

#### Imputation accuracy

Imputation of WGS data is particularly challenging because typically a very large number of variants need to be imputed for a very large number of individuals from few sequenced individuals. As a consequence, genotype uncertainty can be high [19, 25, 65, 66]. Accuracy of the imputed WGS data is one of the main factors that may limit its potential for genomic prediction. In a simulation study, van den Berg et al. [25] quantified the impact of imputation errors on genomic prediction accuracy and showed that prediction accuracy decreases as errors accumulate, especially in the testing set.

We have assessed the accuracy of our imputation approach elsewhere [38, 52] and recommended that ~ 2% of the population should be sequenced in intensely selected populations. In our study, line D was the line for which genomic prediction accuracy using Top40k performed the worst compared to the marker array. In this line, only 0.9% of the individuals in the population had been sequenced and therefore lower imputation accuracy could be expected. Although there was not enough evidence for establishing a link between sequencing effort and genomic prediction accuracy, we recommend cautious design of a sequencing strategy that is suited to the intended imputation method [52].

Genomic prediction accuracy could be improved by accounting for genotype uncertainty of the imputed WGS. In that case, it could be advantageous to use allele dosages rather than best-guess genotypes [66], although most current implementations of genomic prediction methods cannot handle such information.

#### Preselection of predictor variants

Using WGS data to simply increase the number of variants in the genomic prediction analyses does not improve genomic prediction accuracy [16, 19, 22]. Because of the large number of variants in WGS data, there is a need to remove uninformative variants [22, 30, 62, 65, 67]. The most predictive variants are expected to be variants that are causal or that are at least informative about the causal variants, which depends on their distance to the causal variants [68]. Variants that are in weak linkage disequilibrium with causal mutations have a ‘dilution’ effect, i.e., they add noise and can limit prediction accuracy [22, 30, 67]. However, if too stringent filters are applied during preselection of predictor variants, true causal variants may be removed, which would reduce persistence of accuracy across generations and across populations [62, 69]. For instance, the impact of removing variants with a low minor allele frequency depends on the minor allele frequency of the causal variants and the distance between preselected and causal variants [68]. Removing causal or informative variants is expected to negatively affect multi-line or multi-breed prediction.

A popular strategy to preselect variants for the prediction model is based on association tests. Genome-wide association studies on WGS data are expected to confirm associations that were already detected with marker arrays but also identify novel associations (e.g., [35, 70]; (see Additional file 11: Fig. S10)). However, our empirical GWAS results illustrate how the noise of GWAS with WGS data limits the fine-mapping of the associated regions and the preselection of variants for genomic prediction due to the pervasiveness of linkage disequilibrium (see Additional file 11: Fig. S10) and how GWAS with WGS data can be affected by false positives in a more severe way than GWAS with marker arrays, especially for highly polygenic traits (as shown by results using simulated traits (see Additional file 12: Table S2).

Multi-breed GWAS [4] and meta-analyses [71] are suitable alternatives for reaching much larger population sizes and for combining results of populations with diverse genetic backgrounds. Multi-breed GWAS can be more efficient to identify informative variants than single-breed GWAS, which may benefit even prediction within lines [72]. Because the signal of some variants may go undetected for some traits but not for other correlated traits, combining GWAS results from several traits can also help to identify weak or moderate associations [23]. We did not test whether combining the significant markers from the different single-trait GWAS yielded greater improvements in prediction accuracy [28, 31]. Multi-trait GWAS could be more suited for that purpose [70, 73]. To improve fine-mapping, other GWAS models that incorporate biological information have been proposed (e.g., functional annotation [74] or metabolomics [75]).

Several methods to improve variant preselection for genomic prediction have been proposed. VanRaden et al. [29] suggested that preselecting variants based on the genetic variance that they contribute rather than the significance of the association could be beneficial, because the former would indirectly preselect variants with a higher minor allele frequency. Other authors proposed preselection of variants using statistics such as the fixation index (F_ST_) between groups of individuals with high and low phenotype values to avoid the negative impact of spurious associations [67].

#### Genomic prediction models and methods

It is also likely that genomic prediction models, estimation methods, and their implementations need to be improved to leverage the potential of WGS data. This is an active area of research and multiple novel methodologies have been proposed over the last years. Some examples are a combination of subsampling and Gibbs sampling [76] and a model that simultaneously fits a GBLUP term for a polygenic effect and a BayesC term for variants with large effects based on the model (BayesGC) [24]. Testing alternative models and methods for genomic prediction was outside the scope of this study. However, together with refinements in the preselection of variants, it remains an interesting avenue for further optimisation of the analysis pipeline for WGS data.

Some of the most promising new methods for genomic prediction incorporate prior biological information into the models. One such method is BayesRC [21], which extends BayesR by estimating the proportion of variants effects that are drawn from each normal distribution separately for each of several classes of variants defined based on a priori biological information or other criteria [17, 20]. Similarly, genomic feature BLUP (GFBLUP) [77] could be used to incorporate prior biological information from either QTL databases or GWAS as genomic features [19, 34, 65]. The multi-breed multi-genomic relationship matrices genomic prediction model (MBMG) [26], which fits two genomic relationship matrices according to prior biological information, has also been proposed for multi-breed scenarios to improve genomic prediction in small populations. Finally, haplotype-based models have also been shown to provide greater prediction accuracy with WGS data than variant-based models in pigs [78] and cattle [79]. However, the uptake of such models has been limited so far due to additional complexity of, for example, defining haplotype blocks.

### Suitability of whole-genome sequence data for genomic prediction

The small improvements in genomic prediction accuracy that we achieved with WGS data reflect the limited dimensionality of genomic information in intensely selected livestock populations [61], which typically have small effective population sizes, such that marker arrays already capture a large proportion of their independent chromosome segments. Thus, the use of WGS data in current implementations of genomic prediction should be carefully evaluated against its cost, especially given the large size of the WGS datasets that are required. Sequencing costs are expected to continue to decrease and therefore large WGS datasets will become more affordable in time, while efforts to develop and optimise scalable and accurate pipelines for WGS-based data generation, storage, and analysis are on-going (e.g., [80, 81]). These advances, together with a finer knowledge of the genetic architecture of traits empowered by WGS, could allow a case-by-case refinement of genomic prediction. However, to date, the low robustness of the results for complex traits discourages the generalised use of WGS data for traits that are already accurately predicted by conventional means.

## Conclusions

Our results show that the use of WGS data has some potential to improve genomic prediction accuracy compared to marker arrays in intensely selected pig lines. However, the prediction accuracy with a given set of preselected WGS variants was not robust across traits and lines and the improvements in prediction accuracy compared to marker arrays were generally small. The most favourable results for WGS were obtained when the largest training sets were available and used to preselect variants with statistically significant associations to the trait for augmenting the established marker array. With this method and training sets of around 80k individuals, average improvements of genomic prediction accuracy of 0.025 were observed in within-line scenarios. A combination of larger training sets and optimised pipelines for generating and analysing WGS datasets could further improve genomic prediction accuracy with the use of WGS data. The whole strategy for generating WGS data at the population level must be carefully stress-tested and further optimised. However, with the current implementations of genomic prediction, the use of WGS should be carefully evaluated on a case-by-case basis against the cost of generating WGS data on a large scale.

## Supplementary Information

**Additional file 1: Figure S1.** Population structure of the sequenced pigs according to the two first principal components.

**Additional file 2: Figure S2.** Prediction accuracy for all traits and lines.

**Additional file 3: Figure S3.** Genomic prediction accuracy obtained with each set of preselected whole-genome sequence (WGS) variants (either Top40k or ChipPlusSign) against that obtained with the marker array (Chip) in each of the five replicates.

**Additional file 4: Table S1.** Comparison of the results obtained with a single replicate or with five replicates.

**Additional file 5: Figure S4.** Genomic prediction accuracy by density of preselected whole-genome sequence variants in the prediction model.

**Additional file 6: Figure S5.** Genomic prediction accuracy of whole-genome sequence data in multi-line scenarios compared to the marker arrays in within-line scenarios.

**Additional file 7: Figure S6.** Genomic prediction accuracy of ML-Top40k.

Additional file 8. Genomic prediction and genome-wide association study using simulated traits.

**Additional file 9: Figures S7 and S8.** Genomic prediction results using simulated traits.

**Additional file 10: Figure S9.** Genomic prediction accuracy and bias when preselection of variants and training of the predictive equation are performed in two mutually exclusive subsets.

**Additional file 11: Figure S10.** Genome-wide association study on backfat thickness with marker array or whole-genome sequence data in line A.

**Additional file 12: Table S2.** Genome-wide association study results using simulated traits.

## Data Availability

The software packages AlphaPhase, AlphaImpute, and AlphaPeel are available from https://github.com/AlphaGenes. The software package AlphaSeqOpt is available from the AlphaGenes website (http://www.alphagenes.roslin.ed.ac.uk). The datasets generated and analysed in this study are derived from the PIC breeding programme and not publicly available.
