# Supplementary material for: Genomic prediction with whole-genome sequence data in intensely selected pig lines
Source: Genet Sel Evol. 2022 Sep 24;54:65. doi: 10.1186/s12711-022-00756-0 (PMC9509613; doi:10.1186/s12711-022-00756-0)
Supplement: Supplementary file 1 — Additional file 1: Figure S1. Population structure of the sequenced pigs according to the two first principal components. [file 12711_2022_756_MOESM1_ESM.pdf]

Additional File 1

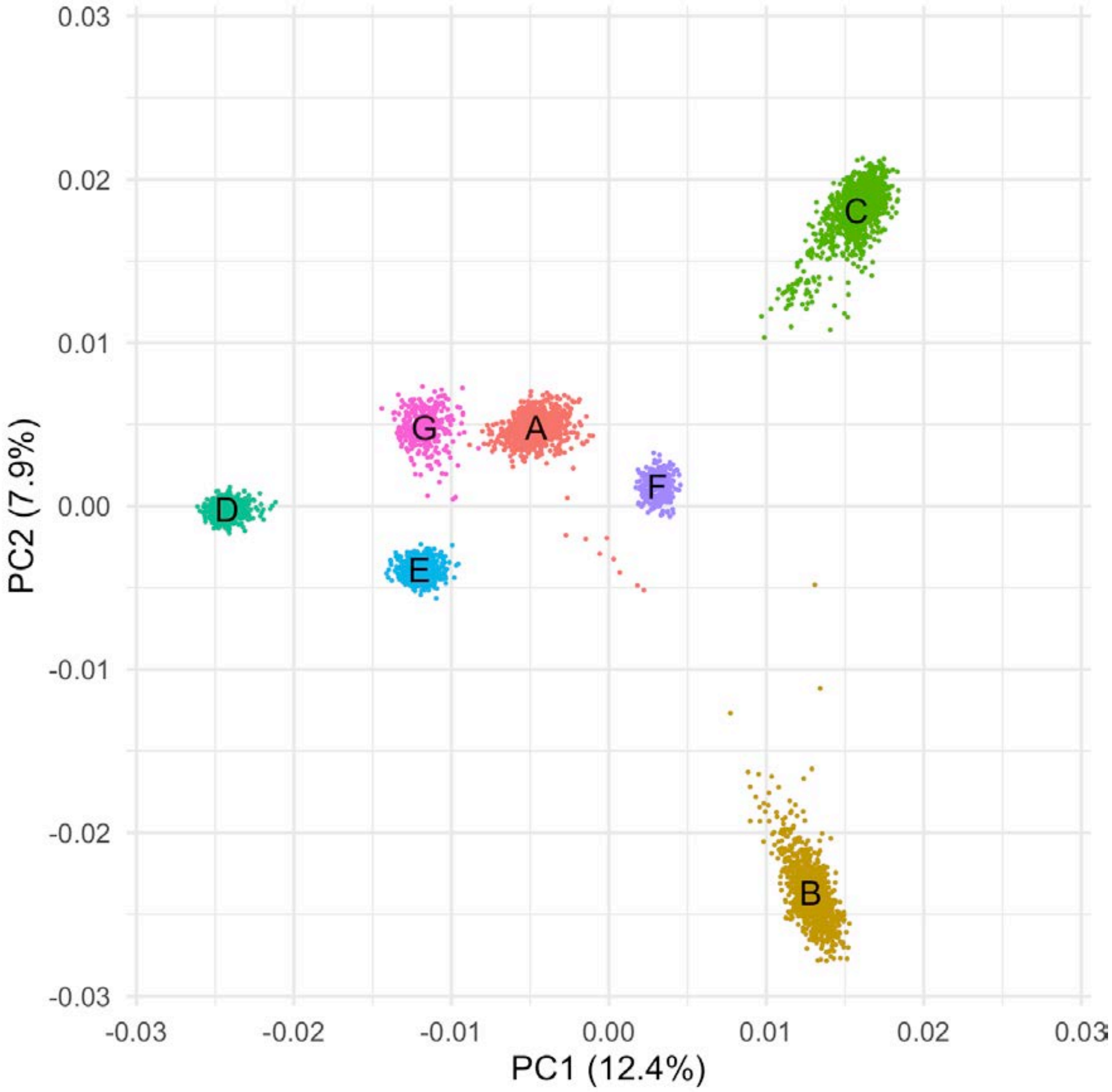

Figure S1. Population structure of the sequenced pigs according to the two first principal components. The colour clusters correspond to lines A to G.
