# Supplementary material for: Genomic prediction with whole-genome sequence data in intensely selected pig lines
Source: Genet Sel Evol. 2022 Sep 24;54:65. doi: 10.1186/s12711-022-00756-0 (PMC9509613; doi:10.1186/s12711-022-00756-0)
Supplement: Supplementary file 3 — Additional file 3: Figure S3. Genomic prediction accuracy obtained with each set of preselected whole-genome sequence (WGS) variants (either Top40k or ChipPlusSign) against that obtained with the marker array (Chip) in each of the five replicates. [file 12711_2022_756_MOESM3_ESM.pdf]

Additional File 3

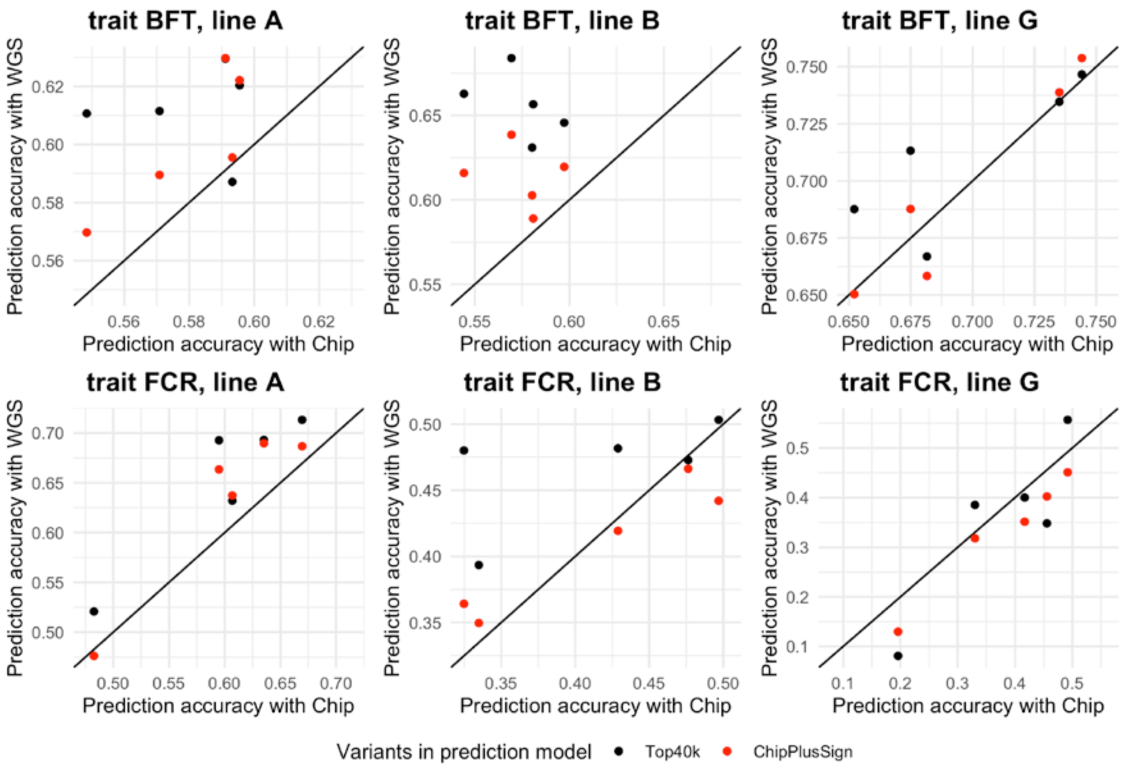

Figure S3. Genomic prediction accuracy obtained with each set of preselected whole-genome sequence (WGS) variants (either Top40k or ChipPlusSign) against that obtained with the marker array (Chip) in each of the five replicates.
