# Supplementary material for: Genomic prediction with whole-genome sequence data in intensely selected pig lines
Source: Genet Sel Evol. 2022 Sep 24;54:65. doi: 10.1186/s12711-022-00756-0 (PMC9509613; doi:10.1186/s12711-022-00756-0)
Supplement: Supplementary file 4 — Additional file 4: Table S1. Comparison of the results obtained with a single replicate or with five replicates. [file 12711_2022_756_MOESM4_ESM.pdf]

Additional File 4

Table S1. Comparison of the results obtained with a single replicate or with five replicates. The difference of the genomic prediction accuracy between each set of preselected whole-genome sequence variants (either Top40k or ChipPlusSign) and the marker array (Chip) is reported.

Trait	Line	Top40k – Chip			ChipPlusSign – Chip		
		Single replicate	Five replicates		Single replicate	Five replicates	
			Mean	SD		Mean	SD
BFT	A	0.042	0.032	0.025	0.025	0.021	0.013
BFT	B	0.054	0.082	0.034	0.043	0.039	0.030
BFT	G	0.011	0.012	0.023	0.002	0.000	0.014
FCR	A	0.040	0.053	0.028	0.030	0.033	0.030
FCR	B	0.065	0.054	0.063	0.009	-0.004	0.035
FCR	G	-0.020	-0.024	0.086	-0.044	-0.048	0.022
