# Supplementary material for: Genomic prediction with whole-genome sequence data in intensely selected pig lines
Source: Genet Sel Evol. 2022 Sep 24;54:65. doi: 10.1186/s12711-022-00756-0 (PMC9509613; doi:10.1186/s12711-022-00756-0)
Supplement: Supplementary file 6 — Additional file 6: Figure S5. Genomic prediction accuracy of whole-genome sequence data in multi-line scenarios compared to the marker arrays in within-line scenarios. [file 12711_2022_756_MOESM6_ESM.pdf]

Additional File 6: Figure S5

Genomic prediction accuracy of whole-genome sequence data in multi-line scenarios compared to the marker arrays in within-line scenarios

<u>ML-ChipPlusSign</u>

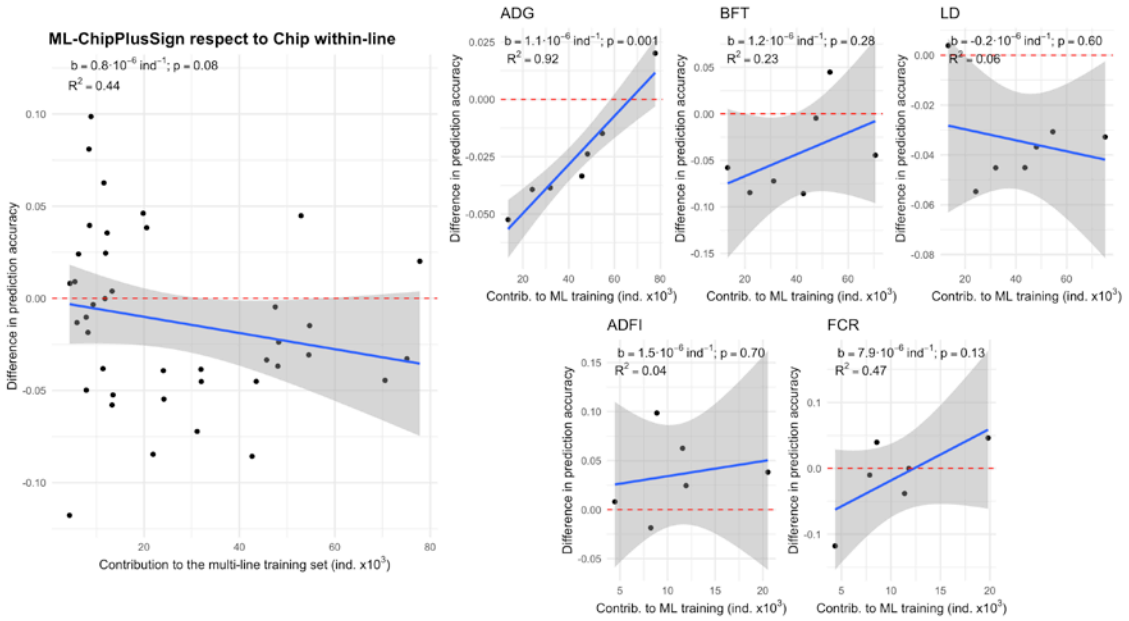

Difference of prediction accuracy between ML-ChipPlusSign and Chip in the within-line scenarios, for all traits and lines (left) or by trait (right). Red dashed line at ‘no difference’. Regression coefficient (b) and p-value of training set size is provided, as well as the coefficient of determination (R^2) of the model. The linear model for the joint analyses included the trait effect.

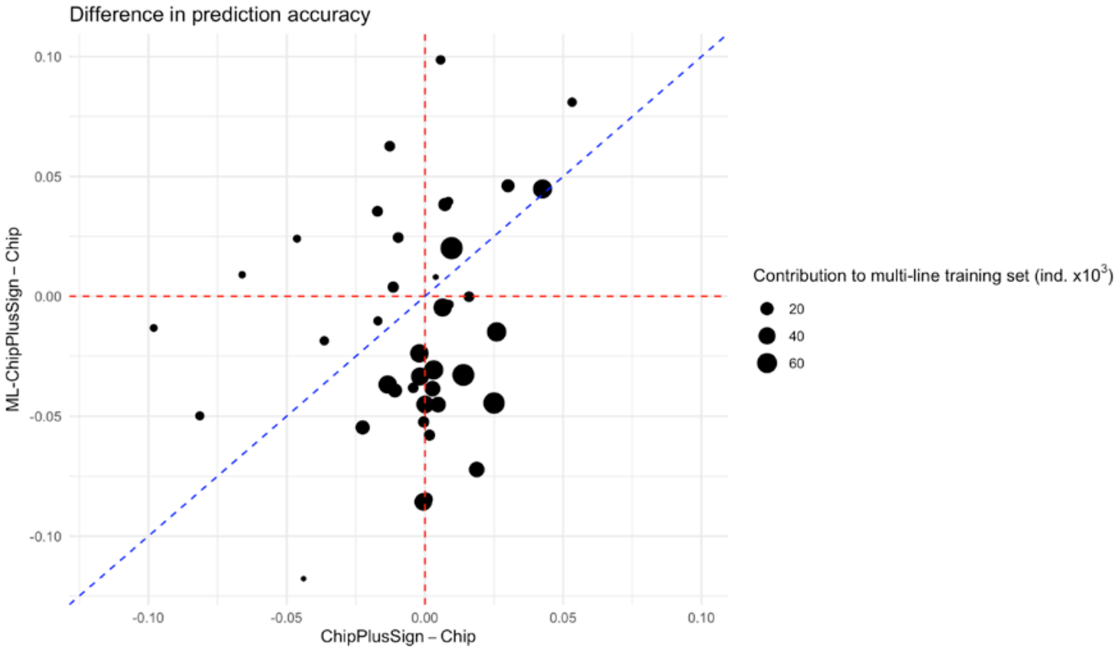

Comparison of the difference in genomic prediction accuracy in the multi-line scenarios (ML-ChipPlusSign) and in the within-line scenarios (ChipPlusSign), both respect to Chip in the within-line scenarios. Red dashed line at 1. Blue dashed line is the bisector.

ML-Top40k

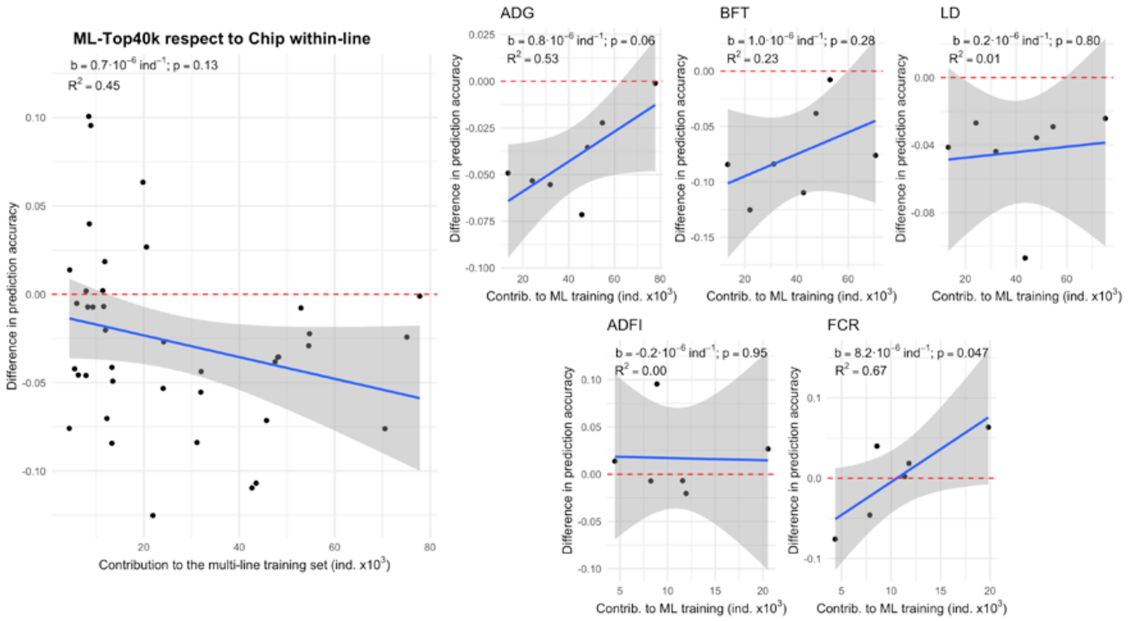

Difference of prediction accuracy between ML-Top40k and Chip in the within-line scenarios, for all traits and lines (left) or by trait (right). Red dashed line at ‘no difference’. Regression coefficient (b) and p-value of training set size is provided, as well as the coefficient of determination (R^2) of the model. The linear model for the joint analyses included the trait effect.

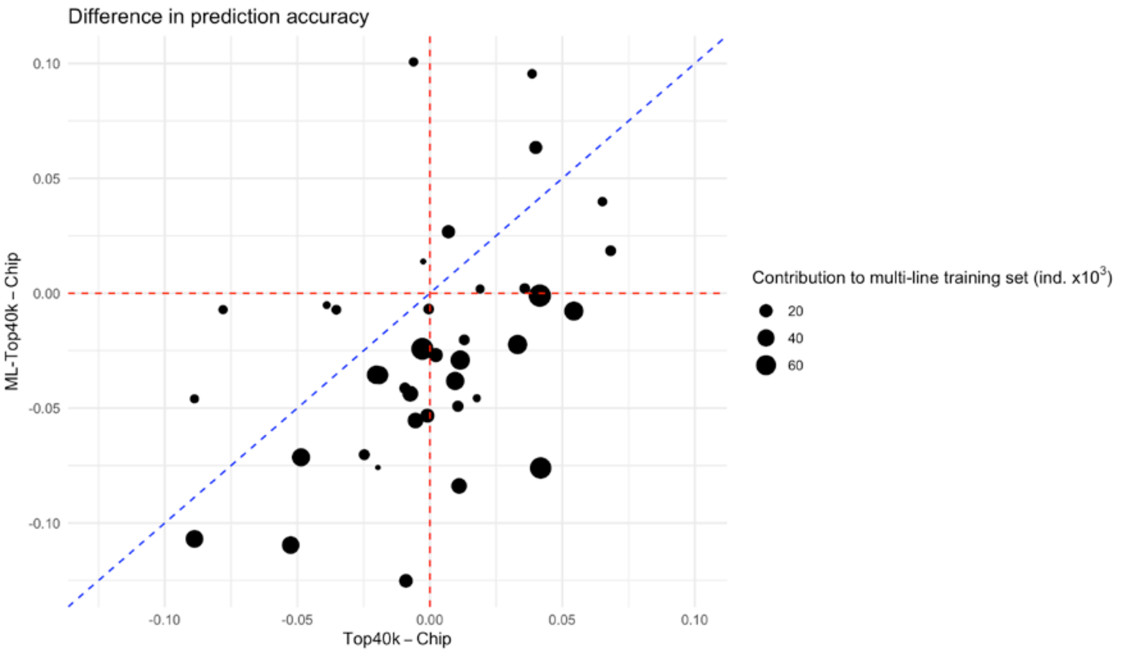

Comparison of the difference in genomic prediction accuracy in the multi-line scenarios (ML-Top40k) and in the within-line scenarios (Top40k), both respect to Chip in the within-line scenarios. Red dashed line at 1. Blue dashed line is the bisector.
