# Supplementary material for: Genomic prediction with whole-genome sequence data in intensely selected pig lines
Source: Genet Sel Evol. 2022 Sep 24;54:65. doi: 10.1186/s12711-022-00756-0 (PMC9509613; doi:10.1186/s12711-022-00756-0)
Supplement: Supplementary file 7 — Additional file 7: Figure S6. Genomic prediction accuracy of ML-Top40k. [file 12711_2022_756_MOESM7_ESM.pdf]

Additional File 7: Figure S6

Genomic prediction accuracy of ML-Top40k

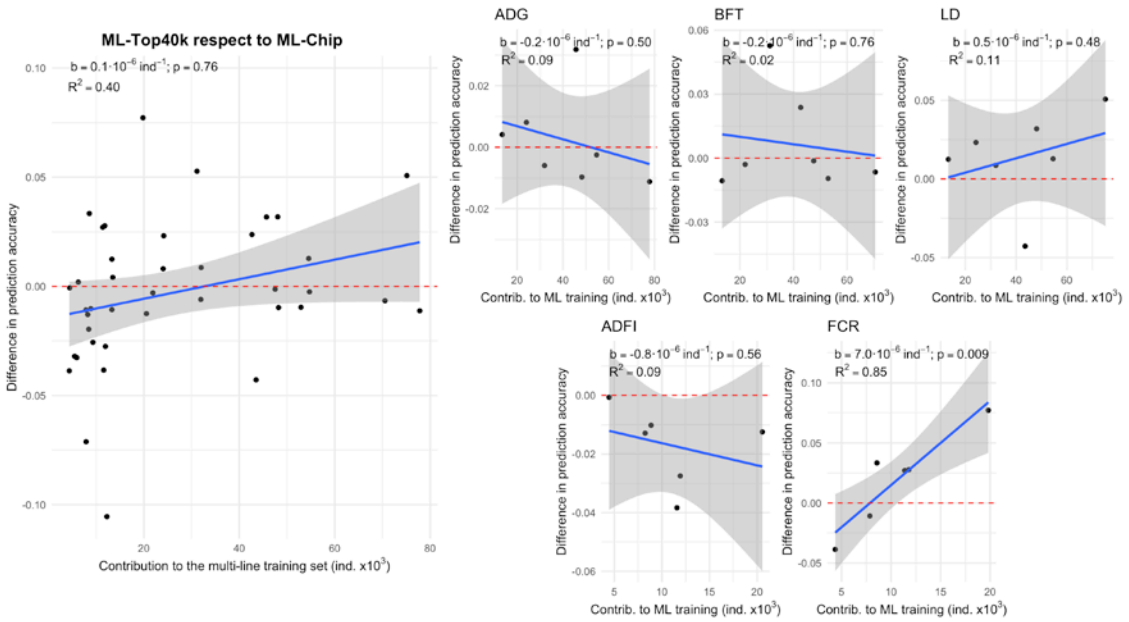

Difference of prediction accuracy between ML-Top40k and ML-Chip in the within-line scenarios, for all traits and lines (left) or by trait (right). Red dashed line at ‘no difference’. Regression coefficient (b) and p-value of training set size is provided, as well as the coefficient of determination (R^2) of the model. The linear model for the joint analyses included the trait effect.

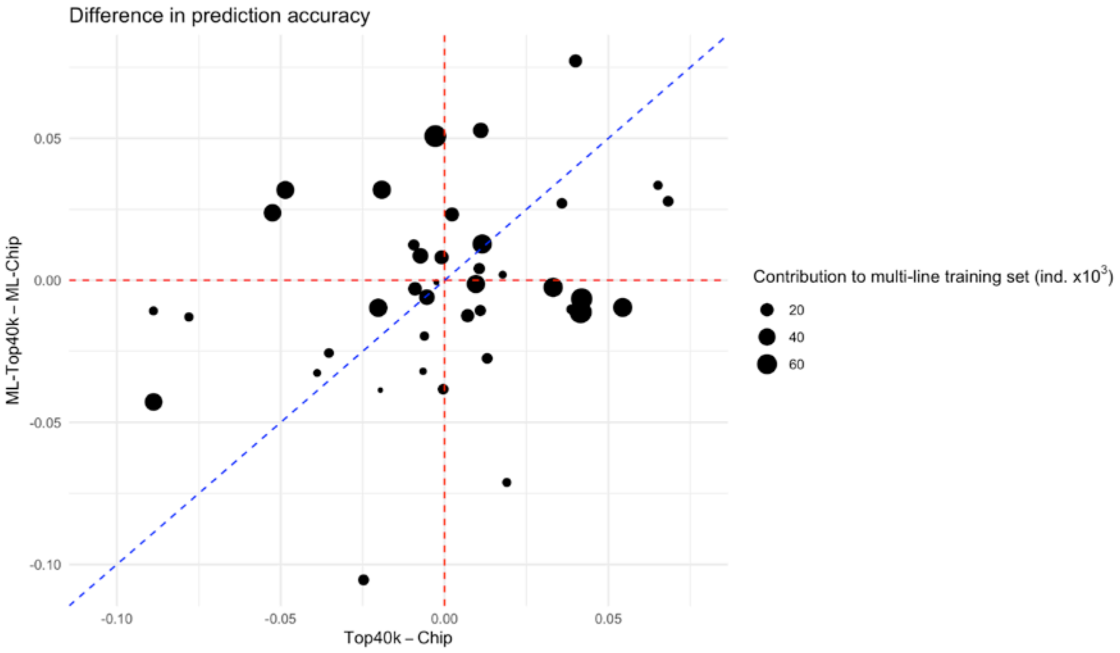

Comparison of the difference in genomic prediction accuracy in the multi-line scenarios (between ML-Top40k and ML-Chip) and in the within-line scenarios (between Top40k and Chip). Red dashed line at ‘no difference’. Blue dashed line is the bisector.
