# Supplementary material for: Genomic prediction with whole-genome sequence data in intensely selected pig lines
Source: Genet Sel Evol. 2022 Sep 24;54:65. doi: 10.1186/s12711-022-00756-0 (PMC9509613; doi:10.1186/s12711-022-00756-0)
Supplement: Supplementary file 8 — Additional file 8. Genomic prediction and genome-wide association study using simulated traits. [file 12711_2022_756_MOESM8_ESM.pdf]

Additional file 8: Supplementary Methods

Genomic prediction and genome-wide association study using simulated traits

To assist the interpretation of the empirical results, we simulated nine traits with different numbers of quantitative trait nucleotides (QTN; 100, 1,000 or 10,000 QTN) and heritability levels (h^2; 0.10, 0.25 or 0.50). Positions of the QTN were sampled randomly amongst all variants called across all lines. Because QTN were sampled from all variants, some QTN were fixed in some of the lines while segregating in others. There were only negligible differences in the number of segregating QTN per line (53 to 61, 531 to 583, or 5375 to 6058, respectively). Effects of the QTN were sampled from a gamma distribution with shape of 2 and scale of 5. We calculated the breeding value for each individual using these QTN genotypes and effects. We then added a residual term sampled from a normal distribution with a variance parameter adjusted to produce a phenotype with the targeted heritability. A phenotype value was calculated for every individual with imputed genotypes that passed the imputation accuracy control (104,661 to 17,224 individuals per line; see Table 1 in the main text). In these simulations, we used the imputed genotypes as real genotypes and, therefore, implicitly cancelled any errors that might arise from the processing of the sequencing reads and genotype imputation.

Further analyses including GWAS and genomic prediction were performed as described in the main text for the empirical traits. Because of the high computational demands of these analyses, no repetitions were performed and therefore interpretation of these results should be cautious.
