# Supplementary material for: Genomic prediction with whole-genome sequence data in intensely selected pig lines
Source: Genet Sel Evol. 2022 Sep 24;54:65. doi: 10.1186/s12711-022-00756-0 (PMC9509613; doi:10.1186/s12711-022-00756-0)
Supplement: Supplementary file 9 — Additional file 9: Figures S7 and S8. Genomic prediction results using simulated traits. [file 12711_2022_756_MOESM9_ESM.pdf]

Additional file 9: Figures S7 and S8

Genomic prediction results using simulated traits

The improvements of genomic prediction accuracy of whole-genome sequence data (WGS) compared to the marker array depended on the genetic architecture of the traits. Traits with high heritability and low number of QTN were more likely to show larger improvements in prediction accuracy. With Top40k (Figure S7), heritability seemed to be the main factor that affected the expected improvement with large training sets (from null improvements when h^2=0.1 to improvements of approximately 0.05 when h^2=0.5, regardless of the number of QTN, with a training set of 92k individuals). With ChipPlusSign (Figure S8), the expected improvements with the same training set (92k individuals) were not only greater in magnitude but depended on both heritability and the number of QTN (from null improvements when h^2=0.1 to improvements of approximately 0.03 to 0.10 when h^2=0.5 with a number of 100 to 10k QTN, respectively). Results confirmed the trends observed for the empirical traits; for instance, the higher robustness of ChipPlusSign compared to Top40k.

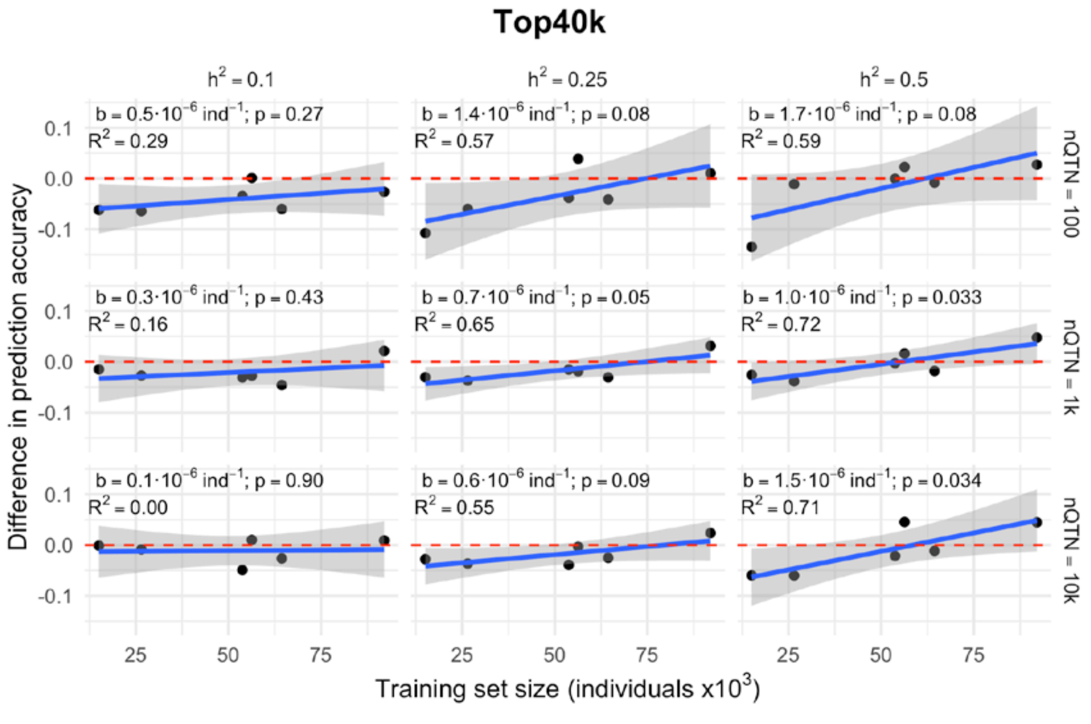

Figure S7. Genomic prediction accuracy with the Top40k variants for the simulated traits. The difference between the Top40k and marker array is shown by heritability (h^2) and number of quantitative trait nucleotides (nQTN) of the simulated traits. Red dashed line at 'no difference'. Regression coefficient (b) and p-value of training set size is provided, as well as the coefficient of determination (R^2) of the model.

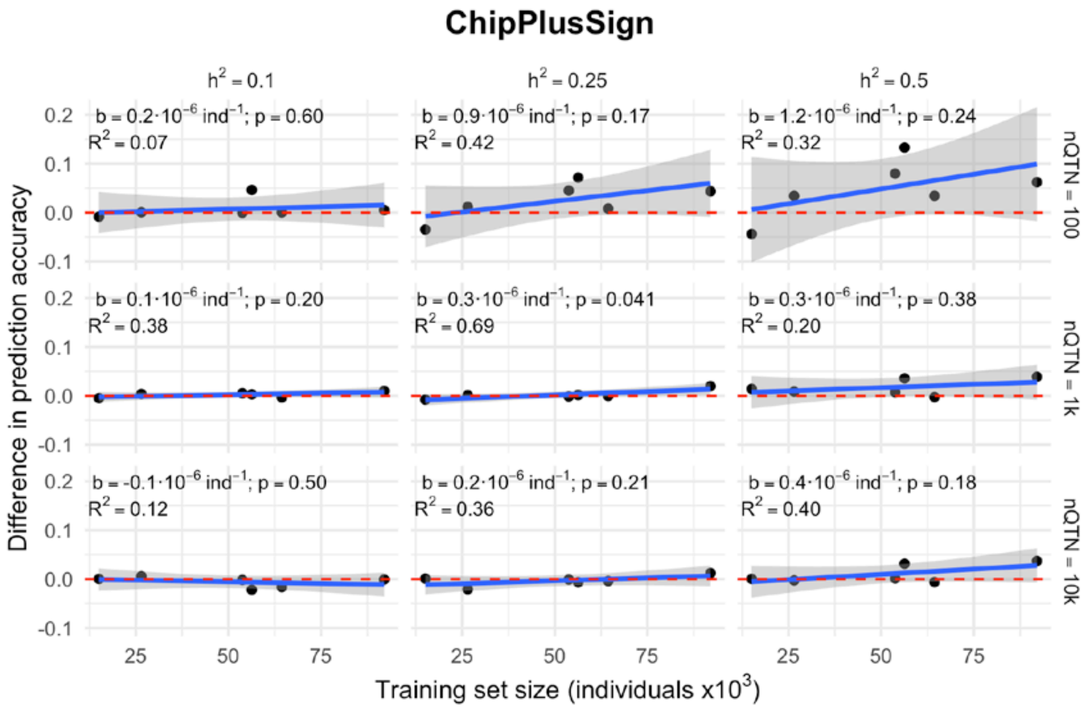

Figure S8. Genomic prediction accuracy with the ChipPlusSign variants for the simulated traits. The difference between the ChipPlusSign and marker array is shown by heritability (h^2) and number of quantitative trait nucleotides (nQTN) of the simulated traits. Other details as in Figure S7.
