# Supplementary material for: Genomic prediction with whole-genome sequence data in intensely selected pig lines
Source: Genet Sel Evol. 2022 Sep 24;54:65. doi: 10.1186/s12711-022-00756-0 (PMC9509613; doi:10.1186/s12711-022-00756-0)
Supplement: Supplementary file 12 — Additional file 12: Table S2. Genome-wide association study results using simulated traits. [file 12711_2022_756_MOESM12_ESM.pdf]

Additional file 12: Table S2

Genome-wide association study results using simulated traits

We defined genomic regions that contained significant associations by overlapping 500-kb segments centred on the significant markers ($p \leq 10^{-6}$). Table S2 shows the number of regions with significant associations that were detected using either the marker array or WGS, and whether they contained zero, one or multiple QTN. The WGS detected a much larger proportion of QTN than the marker array, especially for the traits with high heritability and with large population sizes. The most favourable scenarios for identifying regions that contained unequivocally a single QTN with WGS were those in which the trait was controlled by a low number of QTN. However, even though the genetic architecture was very simple and consisted only of additive effects, the regions with significant associations only captured a small fraction of the QTN that segregated within each line. Moreover, using WGS also increased the number of regions with significant associations that contained no QTN, which could therefore be considered as false positives. Some of the selected regions contained multiple QTN, which could indicate either a 'hit by chance' or an inability to disentangle multiple causal variants. While false positives also occur with marker array, their incidence was more severe with the WGS, especially for traits with a large number of QTN. Large population sizes further aggravated the inflation of genome-wide p-values.

Table S2. Number of genomic regions around significantly associated markers that contained 0, 1 or 2 or more quantitative trait nucleotides (QTN).

h^2	nQTN	Line size	Marker array		Whole-genome sequence		
			0 QTN	1 QTN	0 QTN	1 QTN	≥2 QTN
0.10	100	27k	4	1	8	6	0
		56k	11	3	19	19	0
		92k	10	7	44	19	0
	1k	27k	1	0	4	0	1
		56k	1	0	16	3	1
		92k	1	0	283	9	0
	10k	27k	1	0	1	0	0
		56k	0	0	16	2	1
		92k	2	0	186	17	12
0.25	100	27k	11	6	26	15	1
		56k	22	8	44	28	3
		92k	20	7	90	34	1
	1k	27k	0	0	8	1	3
		56k	3	0	34	15	6
		92k	6	0	692	49	16
	10k	27k	0	0	2	0	0
		56k	0	0	90	9	22
		92k	4	0	564	56	164
0.50	100	27k	18	9	24	24	1
		56k	30	13	116	41	3
		92k	17	9	425	44	1
	1k	27k	6	0	22	9	6
		56k	5	1	238	59	32
		92k	11	1	903	169	120
	10k	27k	0	0	4	0	0
		56k	0	0	360	77	172
		92k	10	0	379	116	508
